# The Role of PPARs in Breast Cancer

**DOI:** 10.3390/cells12010130

**Published:** 2022-12-28

**Authors:** Binggong Zhao, Zhiqiang Xin, Ping Ren, Huijian Wu

**Affiliations:** 1 Key Laboratory of Protein Modification and Disease, School of Bioengineering, Dalian University of Technology, Dalian 116024, China; 2 Hospital Office, The Second Hospital of Dalian Medical University, Dalian 116023, China

**Keywords:** breast cancer, PPARs, ligands, ERs

## Abstract

**Simple Summary:**

Breast cancer is a highly malignant tumor that threatens the health of women worldwide, with extremely high morbidity and mortality. The study of the related genes that affect the occurrence and development of breast cancer can provide more clinical evidence for its prevention and treatment. Peroxisome proliferators-activated receptors are a class of ligand-dependent nuclear receptor transcription factors discovered in 1990 that can regulate the transcription of many genes involved in various cellular physiological processes. The dysregulation of these physiological processes is highly correlated with the occurrence of various diseases, including malignant tumors. Additionally, a large number of reports have indicated that the transcriptional regulation function of peroxisome proliferator-activated receptors and its abnormal expression are related to breast cancer. This article summarizes the role of peroxisome proliferator-activated receptors and their different ligands in the progression of breast cancer since their discovery by searching relevant literature. The purpose of this review is to regard peroxisome proliferators-activated receptors as the new targets for the prevention of breast cancer and to incorporate their ligands into the new evidence for clinical drug combination therapy, especially for high-recurrence triple-negative breast cancer.

**Abstract:**

Breast cancer is a malignant tumor with high morbidity and lethality. Its pathogenesis is related to the abnormal expression of many genes. The peroxisome proliferator-activated receptors (PPARs) are a class of ligand-dependent transcription factors in the nuclear receptor superfamily. They can regulate the transcription of a large number of target genes, which are involved in life activities such as cell proliferation, differentiation, metabolism, and apoptosis, and regulate physiological processes such as glucose metabolism, lipid metabolism, inflammation, and wound healing. Further, the changes in its expression are associated with various diseases, including breast cancer. The experimental reports related to “PPAR” and “breast cancer” were retrieved from PubMed since the discovery of PPARs and summarized in this paper. This review (1) analyzed the roles and potential molecular mechanisms of non-coordinated and ligand-activated subtypes of PPARs in breast cancer progression; (2) discussed the correlations between PPARs and estrogen receptors (ERs) as the nuclear receptor superfamily; and (3) investigated the interaction between PPARs and key regulators in several signaling pathways. As a result, this paper identifies PPARs as targets for breast cancer prevention and treatment in order to provide more evidence for the synthesis of new drugs targeting PPARs or the search for new drug combination treatments.

## 1. Introduction

Breast cancer is a highly heterogeneous tumor transformed from mammary epithelial cells. For example, it is the most common malignant tumor among female cancer patients worldwide in 2022, with the highest morbidity rate among all cancers (accounting for 31%), second only to lung cancer (15% of all cancer deaths), and the morbidity age tends to be increasingly younger [1]. On the basis of cellular gene expression profiles, 5 subtypes of breast cancer have been defined: luminal type A (ER+/progesterone receptor (PR)+/human epidermal growth factor receptor 2(HER2)-), luminal type B (ER+/PR+/HER2+), HER2-overexpression type (ER-/PR-/HER2+), basal-like type (ER-/PR-/HER2-), and normal-like type (the gene expression profile of cells is similar to that of normal breast epithelial cells, showing features of a low treatable rate via chemotherapy, a high quality prognosis, and a lower mortality rate if detected and treated early) [2,3]. In addition, the pathogenesis and progression of breast cancer are accompanied by the differential expression of many genes. Therefore, investigating the molecular mechanism of breast cancer occurrence and development and identifying valuable clinical markers and new therapeutic targets will contribute to the clinical diagnosis and drug treatment of breast cancer. It is also crucial to reducing the lethality of malignant breast cancer.

PPARs are a class of ligand-dependent nuclear transcription factors in members of the steroid hormone receptor superfamily, discovered in 1990 [4]. It is a biosensor of lipid metabolism changes in organisms, especially intracellular fatty acid levels. In addition, such lipid sensors are also involved in the regulation of cell differentiation, growth, and apoptosis in various cells of the organism. The PPARs are expressed in many species, including all mammals [5]. Moreover, the peroxisome proliferator response element (PPRE) sequences on these gene promoters were bound by the heterodimers of PPARs and retinoid X receptors (RXRs) to regulate downstream genes. In the non-ligand-bound state, the PPAR/RXR heterodimer binds to co-repressors and inhibits target gene transcription. The conformation of PPARs changes once the specific ligands are bound, which allows multicomponent complexes to release co-repressors and recruit co-activators: peroxisome proliferator-activated receptor gamma coactivators (PGCs), steroid receptor coactivators (SRCs), CREB-binding protein/p300 (CBP/p300), etc., and regulate the transcription of genes that participate in various physiological processes [6], such as lipid and glucose metabolism, inflammation, and wound healing. Additionally, the expression changes of these genes are found in many diseases, such as dyslipidemia, obesity, type 2 diabetes, metabolic syndrome, etc. [7,8]. To date, many researchers have reported that PPARs function as key players in various malignancies, including breast cancer. In this paper, we analyzed the role of PPARs in breast cancer progression by retrieving the related experimental articles from PubMed in order to provide more evidence for the prevention and treatment of breast cancer.

## 2. Structure of PPARs

**Figure 1:**
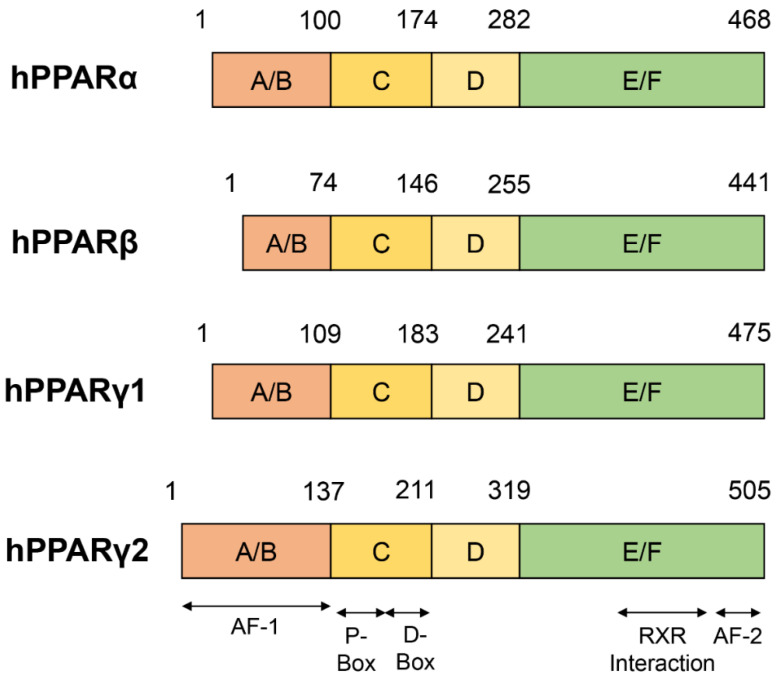
Schematic representation of the principal domains of PPARs. PPARα, PPARβ, and PPARγ all have a modular structure that contains four domains: A/B domain, C domain, D domain, and E/F domain. The A/B domain contains an AF-1 region involved in the regulation of PPARs phosphorylation. The C domain is the DNA binding domain. The D domain is a hinge domain. The E/F domain contains an AF-2 region and is the RXR, ligand, and cofactor binding site.

PPARs comprise three subtypes that have a high degree of homology: PPARα, PPARβ/δ and PPARγ. The PPARs contain a modular structure consisting of an amino-terminal ligand-independent transcriptional activation A/B domain, a 70 amino acid-long DNA-binding C domain, a hinge D domain, and a carboxyl-terminal ligand-binding E/F domain composition (Figure 1) [9,10].The sequence structure of the C and E/F domains of PPARs subtypes has high homology [5].

Furthermore, the transcriptional activation of the A/B domain has phosphorylation-binding sites [11]. The phosphorylation state of this region regulates the affinity of PPARs for receptors (PPRE), ligands, and coactivators and is also a regulatory region used by PPARs to restrict the transcription of most genes [12,13,14]. The A/B domain is a highly variable region containing an activation function-1 (AF-1) domain, which has not been fully characterized. Additionally, the central DNA-binding C domain has two highly conserved C_4_ zinc finger motifs: distal (D-box) and proximal (P-box) boxes, which confer heterodimerization and PPARs DNA binding, respectively. The C domain recognizes and binds to the PPRE motif (AGGTCANAGGTCA) on the promoter sequences of target genes. The hinge D domain supports the conformational change of PPARs upon ligand binding. The ligand-specific E/F domain is a spherical structure composed of 13 α-helices (H1–H12, H2’) and 4 short β-strands (S1–S4) [15]. On the other hand, the anti-parallel α-helical forms a sandwich structure: H3, H7, and H10/H11 form the two outer layers of the sandwich; H4, H5, H8, and H9 form the central layer of the sandwich. The central layer is mostly located in the upper half of the sphere. The lower half of the sphere consists of H3, H5, and H10, forming a very large Y-shaped cavity (~1400° A3). The three-directional arms of the Y-shaped cavity allow PPARs to be ligand-bound with various single-chain or branched structures [16]. The RXR interacts with several α-helices, including H7–H10, to form PPAR/RXR heterodimers. Further, Sheu et al. identified 10 binding “hot spots” for RXRs in PPARγ using solvent mapping techniques. Four of these spots are located within the Y-shaped cavity: two around the entry site of the Y-shaped cavity, two in the coactivator binding region, one in the dimerization domain, and one in the secondary locus [17]. The E/F domain is also a binding site for coactivators and co-repressors. The end of the E/F domain contains a domain called AF-2, which is highly conserved in all subtypes of PPARs and is closely related to the events of ligand-induced transcription. Ligand binding to the E/F domain induces a conformational change in the AF-2 domain, resulting in a suitable binding surface to recruit coactivators and promoting target gene transcription [18]. In addition, studies on the phosphorylation of PPARs have shown that phosphorylation of AF-1 could affect the activity of AF-2, revealing that the activity of PPARs is affected by intramolecular kinase cascade signaling. All domains participate in the physiological activities of PPARs as a unified whole. For example, changes in the A/B domain could affect ligand binding in the E/F domain [19] or DNA binding in the C domain [20].

**Figure 2:**
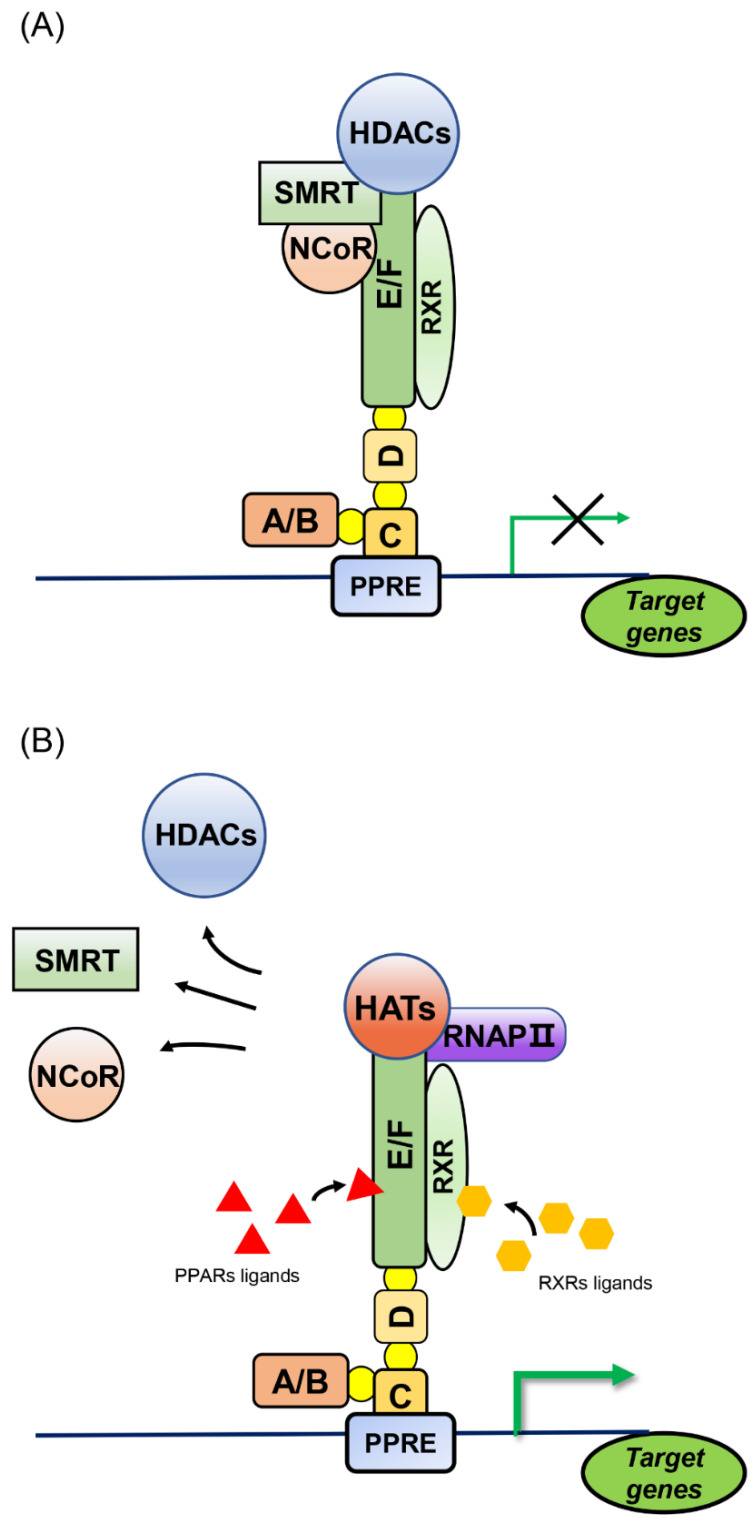
PPARs-mediated gene regulation. PPAR forms a heterodimer with RXR and binds to the PPRE element of the target gene promoter. In the absence of ligand binding, the heterodimer recruits transcriptional corepressors such as NCoR and SMRT, as well as HDACs, to repress target gene transcription (**A**). Upon ligand binding, PPAR changes conformation, releases transcriptional repressor complexes, and recruits transcriptional coactivators such as RNAPII and HATs to promote target gene transcription (**B**). A/B, C, D, E/F: PPAR domains; PPRE: peroxisome proliferator response element; RXR: retinoid X receptor; NCoR: nuclear receptor corepressor 1; SMRT: nuclear receptor corepressor 2; HDACs: histone deacetylases; HATs: histone acetyltransferases; RNAPII: RNA polymerase II.

The heterodimer of PPAR and RXR is considered a permissive dimer because activation of either component can activate the entire complex. The PPAR/RXR heterodimer binds to the target gene promoter, PPRE. In the non-liganded state, PPAR/RXR interacts with co-repressors such as SMRT and NCoR to recruit repressors that contain histone deacetylase (HDAC) activity, thereby inhibiting gene transcription [21]. Upon ligand stimulation, PPAR/RXR dissociates from multicomponent co-repressors, recruits RNA polymerase II and activators with histone acetyltransferase (HAT) activity, remodels chromatin structure, and promotes target gene transcription (Figure 2) [22].

## 3. Ligands for PPARs

The PPARs require ligand activation, such as natural and synthetic ligands, which is a characteristic of many other steroid hormone receptors [4,23,24]. The natural ligands consist of a group of endogenously secreted molecules, including various unsaturated fatty acids and their metabolic products. The specificity and activity of these molecules are not high in most circumstances. Additionally, the incubation of triglyceride-rich lipoproteins with lipoprotein lipase (LPL) produces many ligands for PPARs [25,26]. Certain prostaglandins and their metabolic derivatives are also natural ligands [27]. The structure and geometry of PPARβ/δ and PPARα are similar, while PPARγ more likely to bind long-chain polyunsaturated fatty acids [28]. At present, a variety of synthetic ligands are active on the market. These synthetic ligands often have higher PPAR subtype specificity and stronger metabolic activity than natural ligands. The synthetic ligands include agonists and antagonists (Table 1). The antagonists are also referred to as “inverse agonists” because, although they bind to the agonist binding sites of PPARs, they cause opposite pharmacological responses by stabilizing the binding state of uncoordinated PPARs and multicomponent co-repressors in order to inhibit transcriptions of downstream target genes [29,30]. Physical changes caused by ligand binding include changes in the three-dimensional structure [31,32], dissociation of heat shock proteins and chaperones [33,34], and nuclear entry [35,36] of PPARs.

The ligands of one subtype of PPARs could also act on other subtypes. For example, the natural exogenous fatty acid ombuin-3-O-β-D-glucopyranoside was shown to simultaneously activate PPARα and PPARβ/δ to reduce the expression of the lipogenic genes in hepatocytes and promote the genes’ expression, which are related to reversed cholesterol transportation in macrophages so as to reduce intracellular lipid concentration [37]. This could provide dual agonists or even pan-agonists of PPARs for the clinic. The dual-agonist glitazars targeting PPARα and PPARγ, such as muraglitazar and tesaglitazar, are being tested in clinical trials and are expected to reduce cardiovascular risk. In addition, the lipid-lowering fibrate acid derivative, bezafibrate, is the first pan-agonist of PPARs that has been clinically tested with satisfactory safety levels and has become the reference for pan-agonists of PPARs [38]. Conversely, 13-HODE, an oxidized low-density lipoprotein, acts as a ligand to activate PPARγ [39]. However, it has the opposite results when it acts on PPARβ/δ. For example, when it acts on colorectal cancer cells, it is considered an antagonist that down-regulates the expression of PPARβ/δ and induces tumor cell apoptosis [40]. In preadipocytes, it is considered an agonist, activating PPARβ/δ to protect the liver from chemically induced liver injury [41]. The ligands were shown to be tissue-specific for the biological activity of PPARs, which may be due to the presence or absence of other regulatory factors in addition to known ligands. In fact, long-term bioassay studies have shown that high-affinity dual PPARα/PPARγ agonists could raise clinical safety concerns, including potential carcinogenicity, weight increase, peripheral dropsy, and a potential increased risk of heart failure in rodents [42]. Therefore, the development of dual agonists and pan-agonists of PPARs with relatively low affinity (i.e., µM or nM) is more suitable for cancer chemoprevention [43]. In addition, the use of PPARγ single agonists, thiazolidinediones (TZDs), induces bone loss in postmenopausal females and diabetic patients [44,45,46,47]. In contrast, administration of PPARα and PPARβ/δ dual agonists, linoleic acid (LA), or PPARs pan-agonist bezafibrate could upregulate bone mineral density and result in the formation of periosteal bone in male rats [48]. This suggests that dual and pan-agonists of PPARs have the potential to counteract the adverse effects elicited by the use of highly specific single agonists.

### 3.1. Agonists and Antagonists of PPARα

The most classic agonists of PPARα are fibrates, including bezafibrate, fenofibrate, clofibrate, gemfibrozil, and Wy-14,643 [49]. Wy-14,643, a pirinixic acid first discovered to play an effective role in anti-hypercholesterolemia [50], induces marked hepatomegaly and peroxisome proliferation in hepatocytes and reduces serum cholesterol and triglyceride levels in male mice [51]. Subsequent reports confirmed that Wy-14,643 is a specific activator of PPARα [52]. The Wy-14,643-activated PPARα regulates yes-associated protein (YAP) expression and nuclear translocation, and blockade of YAP signaling abolishes PPARα-induced hepatocyte hypertrophy and hepatocyte proliferation in mice [53]. GW9578, a urea-substituted thioisobutyric acid (TiBA), is a potent murine PPARα-selective agonist, but it has only a 20-fold selectivity for human PPARα [54]. Furthermore, GW9578 exists in the form of a viscous oil or foam, which provides a hindrance to the quantitative treatment of experiments in vitro and in vivo. Brown et al. identified GW7647 through solid-phase array synthesis to aid in identifying PPARα agonists with high selectivity and good physical properties [55]. As a thioisobutyric acid derivative, GW7647 is the first identified PPARα-specific agonist. It has a 200-fold higher specificity than PPARβ/δ and PPARγ and has lipid-lowering activity in vivo. The GW7647 is an excellent PPARα-specific agonist that could be used in experimental research since it is a powder with a melting point of 153–154 °C [28].

**Figure 3:**
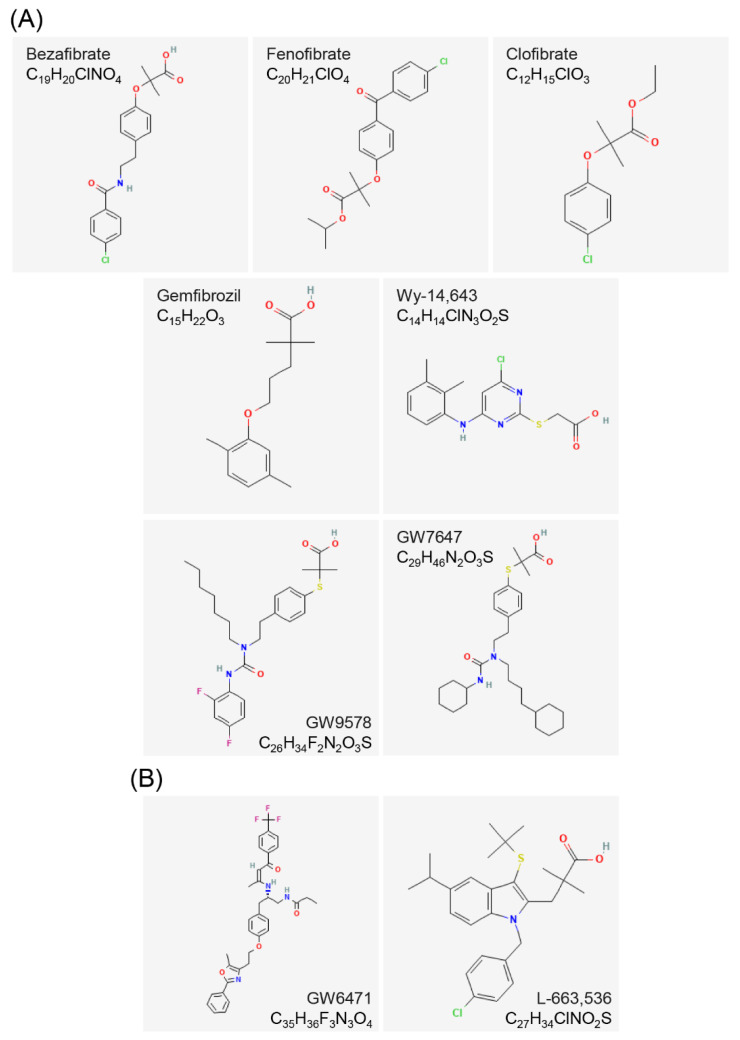
Agonist (**A**) and antagonist (**B**) secondary structures of PPARα.

GW6471 is a specific antagonist of PPARα. GW6471 and PPARα could form a ternary complex with the transcriptional co-repressor SMRT, and GW6471 further strengthens the binding of the PPARα E/F domain to the SMRT co-repression motif. The co-repression motif in the ternary complex adopts a three-turn α-helix, preventing the PPARα AF-2 domain from assuming the active conformation [56]. Additionally, L-663,536 (MK-886), a leukotriene biosynthesis inhibitor, was originally identified to prevent endogenous leukotriene production during allergic reactions in guinea pigs and protect them from lethal anaphylactic shock [57]. However, it was subsequently identified as an inhibitor of the fatty acid binding protein 5-lipoxygenase-activating protein (FLAP), but the ability of L-663,536 to induce apoptosis was not mediated by FLAP [58]. The drug L-663,536 was not identified as a non-competitive antagonist of PPARα until 2001. It was then discovered to prevent the conformational change necessary for the PPARα activity formation and inhibit the PPARα target gene transcriptional activity (Figure 3) [59].

### 3.2. Agonists and Antagonists of PPARβ/δ

The first synthetic agonist was L-165,041 [60]. It is a leukotriene antagonist that can activate both the human PPARβ/δ gene and PPARγ [61]. GW501,516 is a more potent and specific PPARβ/δ agonist [62]. It has been used in a large number of experiments so far and has become the reference for PPARβ/δ agonists [63]. However, it was subsequently reported that GW501,516 had no hepatoprotective and anti-fibrotic effects in patients with chronic liver disease [64]. Further, the GW501,516 has been limited for use in clinical trials due to its potential metabolic derangement and stimulant effects and the high risk of a halt in the evolution of molecules after uncontrolled application [65]. The agonist GW0742, which was developed at the same time as GW501,516, has become a highly selective agonist of PPARβ/δ in commercial non-human experiments [66]. The most clinically used PPARβ/δ agonists are MBX-8025/RWJ80,025 and KD-3010 (Phase II trial) [67,68].

**Figure 4:**
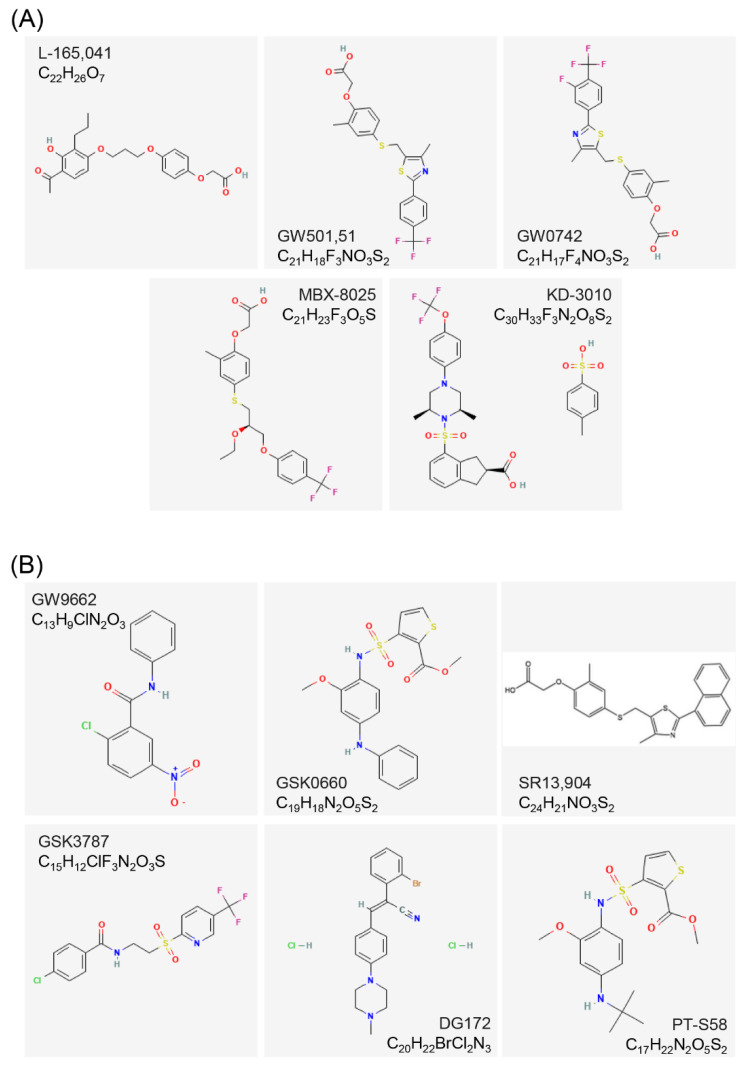
Agonist (**A**) and antagonist (**B**) secondary structures of PPARβ/δ.

The earliest PPARβ/δ antagonist used is an irreversible PPARγ antagonist, GW9662 [69]. In 2008, GSK0660 was confirmed as the first PPARβ/δ selective antagonist [29]. However, due to its low bioavailability, the in vivo experimental effects were affected. On the other hand, SR13,904 is also a PPARβ/δ antagonist, although it also has a weak inhibiting effect on PPARγ [70]. The latest PPARβ/δ antagonist used is GSK3787 which has fair pharmacokinetics. It has been used in a large number of animal experiments due to its fine bioavailability [30,71]. The above compounds are all irreversible antagonists of PPARβ/δ, and covalently bind to the latter. DG172 and PT-S58 are currently two novel PPARβ/δ antagonists. The DG172 has high affinity and strong inhibitory ability. It recruits co-repressors, down-regulates the transcription of PPARβ/δ target genes, and still keeps mice biologically active after oral treatment [72]. In addition, PT-S58 is a cell-permeable diarylcarbonamide drug that acts directly on the PPARβ/δ ligand binding sites. It is a pure competitive specific inhibitor of PPARβ/δ (Figure 4) [73,74].

### 3.3. Agonists and Antagonists of PPARγ

The most typical agonists of PPARγ are TZDs, which were the first high-affinity selective PPARγ agonists identified. The TZD family includes rosiglitazone (RGZ/BRL49,653) [75], pioglitazone (PGZ), ciglitazone (CGZ), troglitazone (TGZ), englitazone (EGZ), and balaglitazone (BGZ). They are all able to specifically activate PPARγ [76]. In addition to their ability to target PPARγ for type 2 diabetes therapy, different TZD compounds are also in clinical trials for their tumor-suppressing effects. They may become anticancer drugs in the near future. The non-TZD ligand of PPARγ, L-764,406, is the first known partial agonist of PPARγ. Covalent binding of L-764,406 to Cys313 of H3 in the E/F domain of PPARγ induces a conformational change in the receptor and specifically activates the transcriptional activity in the receptor [77]. GW0072 is the ligand of PPARγ with high affinity but is a weak partial agonist. It locates in the ligand-binding pocket, which is uncovered by X-ray crystallography, by binding to an epitope distinct from known PPARγ agonists and does not interact with AF-2 [78]. In 1999, it was first discovered that GW7845 (an L-tyrosine derivative) could be used as PPARγ activator to prevent the progression of experimental breast cancer in rats [79].

**Figure 5:**
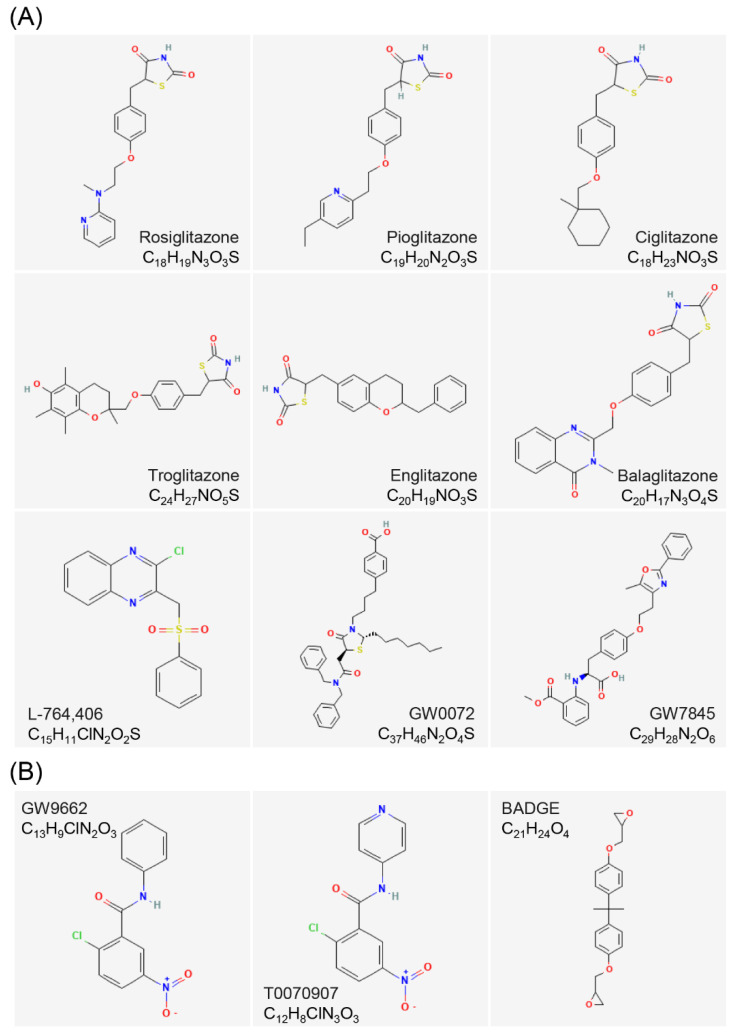
Agonist (**A**) and antagonist (**B**) secondary structures of PPARγ.

GW9662 is an irreversible PPARγ full antagonist [80]. The GW9662 covalently binds to Cys285 of PPARγ, a residue that is highly conserved in all three PPARs. Additionally, GW9662 is 10 to 600 fold more selective for PPARγ than PPARα and PPARβ/δ in cells [81].T0,070,907, which is similar in structure to GW9662, is also a synthetic PPARγ-selective antagonist with more than 800-fold selectivity over PPARα and PPARβ/δ [82]. Bisphenol, a diglycidyl ether (BADGE), also specifically inhibits PPARγ and is a low-affinity PPARγ ligand [83]. The BADGE has been reported to antagonize PPARγ and block adipogenesis induced by BRL49,653 and insulin, under the condition that the concentration level reaches its solubility limit (100 μM) (Figure 5) [84].

**Table 1:** Agonists and antagonists of PPARs.

PPARs	Agonists		Antagonists
PPARα	fibrates	Bezafibrate [49]	GW6471 [56]
		Fenofibrate [49]	L-663,536 [59]
		Clofibrate [49]	
		Gemfibrozil [49]	
		Wy-14,643 [52]	
	GW9578 [54]		
	GW7647 [55]		
PPARβ/δ	L-165,041 [60]		GW9662 [69]
	GW501,516 [62]		GSK0660 [29]
	GW0742 [66]		SR13,904 [70]
	MBX-8025/RWJ80,025 [67]		GSK3787 [71]
	KD-3010 [68]		DG172 [72]
			PT-S58 [73,74]
PPARγ	TZDs	rosiglitazone (RGZ) [75]	GW9662 [80,81]
		pioglitazone(PGZ) [76]	T0,070,907 [82]
		ciglitazone(CGZ) [76]	BADGE [83]
		troglitazone(TGZ) [76]	
		englitazone(EGZ) [76]	
		balaglitazone(BGZ) [76]	
	L-764,406 [77]		
	GW0072 [78]		
	GW7845 [79]		

### 3.4. Structure of PPARs Ligands

The secondary structure of PPARs ligands generally contains fluorine, chlorine, hydroxyl, aliphatic, carboxyl, and carbonyl groups. These groups can form electrophilic groups and interact with relevant sites, such as carboxyl on the E/F domain of PPARs, to form hydrogen bonds and improve the stability of the combination. For example, the carboxyl of the agonist GW409,544 forms a direct hydrogen bond with Try464 on the AF-2 domain of PPARα. GW6471, an antagonist of PPARα, replaces the carboxyl of GW409,544 with an acetamide, destroying the formation of the hydrogen bond on Try464. The GW6471 induces PPARα to recruit SMRT and enhances the binding of PPARα E/F domain to the SMRT co-repression motif, which adopts a three-turn α-helix and prevents the PPARα AF-2 domain from adopting an active conformation [56]. Several ligands contain amino, imino, or quaternary amino groups, which lead to the shift of electrons and form charge attraction with the relevant sites on the Y-shaped cavity of PPARs. The agonist bezafibrate forms a significant positive and negative charge center, which can form a strong salt bond with Lys183 on PPARα [38]. In addition to the above-mentioned intermolecular forces, some ligands can also form covalent bonds with PPARs. Covalent binding of L-764,406 to Cys313 of H3 in the PPARγ E/F domain induces a conformational change in the receptor and specifically activates its transcriptional activity [77]. GW9662, an irreversible full antagonist of PPARγ, covalently binds to Cys285 of PPARγ [81]. In addition, the molecular chains of PPARs agonists are basically long, and most of their electrophilic groups are linked to carbon atoms or small groups. On the contrary, the molecular chains of PPARs antagonists are shorter than those of agonists, and their electrophilic groups are linked to larger carbon rings, aromatic rings, or heterocyclic rings. The antagonists with relatively large molecular structures bind to the ligand-binding cavity of PPARs, resulting in steric hindrance and preventing agonists from entering, thereby inhibiting the active conformational change of PPARs [29,30]. The entrance to the Y-shaped cavity in the PPARs E/F domain includes several polar residues, and the two branches of the cavity, Arm I and Arm II, are mainly composed of hydrophobic residues, except for some moderately polar residues in Arm I. These residues play key roles in determining the interaction of agonists or antagonists with PPARs.

## 4. Subtypes of PPARs and Breast Cancer

The PPARα, PPARβ/δ and PPARγ express differently in different tissues, with differences in target genes, biological activities, and ligand affinities [85]. Among 225 studies of experimentally validated PPAR target genes, 83 genes were PPARα target genes, 83 were PPARβ/δ target genes, and 104 were PPARγ target genes [86]. In fact, the target genes of the three subtypes of PPARs partially overlap. For example, all three PPARs could transcriptionally activate the angiogenesis pathway-related protein Angptl4 and the lipid droplet-associated protein Plin2 after ligand activation [87]. The PPARs participate in the regulation of carbohydrate and lipid metabolism and homeostasis, as well as various physiological processes such as cell differentiation, proliferation, inflammation, and vascular biology [88]. In addition, the three subtypes of PPARs also regulate the occurrence and development of many malignant tumors via different mechanisms; breast cancer is one of them.

### 4.1. PPARα and Breast Cancer

PPARα, the first PPAR identified, is recognized as an orphan receptor activated by a variety of peroxisome proliferators. The PPARα was originally discovered in rodents and was named for its role in peroxisome proliferation [4]. On the other hand, PPARβ/δ and PPARγ were subsequently discovered and identified as cognate receptors that are activated by distinct peroxisome proliferators [24,52]. However, subsequent research proved that all PPARs fail to play a role in human peroxisome proliferation. PPARα is mainly expressed in metabolically vigorous cells with active fatty acid oxidation capacity, for example in skeletal muscle, brown fat, the liver, heart, and intestinal mucosal tissues [89]. PPARα is of considerable importance to glucose and lipid metabolism and the balance of transport in mammals. Its main function of maintaining lipid homeostasis is realized by increasing cell mobilization, promoting cell uptake, activation, oxidation, and decomposition of fatty acids, and generating ketone bodies for energy production [90]. The ligand-activated PPARα could also catalyze the hydroxylation of fatty acids. Hence, PPARα is the target of fibrates and hypolipidemic drugs for the treatment of abnormal lipid metabolism. The transcription of PPARα is up-regulated by fibrates, which enhance the lipolysis mediated by lipoprotein lipase, promote the oxidative decomposition of fatty acids, and achieve the curative effect of reducing total cholesterol and total triglycerides [91]. Fibrates are effective in increasing insulin sensitivity and protecting the cardiovascular system, so they are also widely used in the clinical treatment of diabetes and cardiovascular diseases [92].

In addition to regulating glucose and lipid metabolism, PPARα plays a role in various cancers. Long-term administration of PPARα agonists was reported as early as 1980 to cause liver cancer in rodents [93]. This effect of agonists was dependent on the receptor PPARα, as they (Wy-14,643 or bezafibrate) did not induce liver cancer in PPARα-null mice [94,95]. The pro-hepatocarcinogenesis effect of PPARα agonists was not evident in humans [96]. The species-specific mechanism of promoting hepatocarcinogenesis is that mouse-derived PPARα rather than human-derived PPARα down-regulated let-7C miRNA to increase the stability of its target gene MYC, an oncogenic factor. The increased expression of MYC promoted hepatocyte mitosis until carcinogenesis [97,98,99]. Some studies have shown increased expression of PPARα in endometrial cancer. Fenofibrate treatment significantly prevented the proliferation of endometrial cancer cells and promoted cell apoptosis [100]. However, other studies have also shown that PPARα knockdown inhibited the proliferation of endometrial cancer cells, promoted cell apoptosis, and reduced the secretion of the angiogenesis-related factor VEGF, while fenofibrate treatment also reduced the secretion of VEGF [101]. Since this contradictory phenomenon is not caused by nonspecificity to PPARα and cytotoxicity at the dose of fenofibrate [102], a possible explanation might be the biphasic response of PPARα activity, i.e., PPARα with very low activity and expression and PPARα with very high activity and expression producing the same effect, known as a U-shaped dose-response curve. PPARα was also aberrantly expressed in melanoma. Fenofibrate treatment inhibited the clone formation and migration abilities of melanoma cells and rendered them highly sensitive to staurosporine (a protein kinase C inhibitor with strong pro-apoptotic activity) [103].

Chang et al. found that, compared to adjacent normal tissues, PPARα and its natural ligand, arachidonic acid (AA), were significantly overexpressed in the tissues of breast cancer. The growth of three breast cancer cells, MDA-MB-231 (ER-), MCF7 (ER++++), and BT-474 (ER++), were stimulated by AA, with the most pronounced pro-proliferative effect on MCF7 cells, revealing a positive correlation between PPARα and the proliferation of ER+ breast cancer cells [104]. Human cytochrome P450 1B1 (CYP1B1)-mediated biotransformation of endogenous estrogens and environmental carcinogens promotes the progression of multiple hormone-dependent tumors, including breast cancer [105]. Hwang et al. found that Wy-14,643 increased CYP1B1 mRNA and protein levels in MCF7 cells and activated PPARα enhanced CYP1B1 promoter activity through directly binding to its PPRE elements [106]. In addition, Castelli et al. found that treatment of breast cancer stem cells with the specific PPARα antagonist GW6471 reduced cell proliferation, viability, and spheroid formation, resulting in metabolic dysfunction and apoptosis [107]. The above experiments in vitro all suggest that PPARα functions in promoting the development of breast cancer. However, Pighetti et al. found that treatment with Wy-14,643 inhibited the ability of DMBA to induce breast tumor formation in rats and induced tumor volume regression [108]. Chandran et al. showed that clofibrate treatment activated the PPARα transcriptional activity and exerted an anti-proliferative effect on breast cancer cells by regulating the levels of tumor suppressors, cell cycle inhibitors, and cell to cycle checkpoint kinases, causing cells to arrest in the G0/G1 phase and significantly inhibiting cell growth. In addition, activated PPARα reduced the expression of inflammatory pathway-related enzymes and their receptors, reduced the protein levels of lipogenic enzymes, regulated the fatty acid oxidation associated gene expression, and affected various lipid metabolism pathways [109]. Yin et al. found that Runt-related transcription factor 2 (RUNX2), with high expression in breast cancer, recruited metastasis-associated 1 (MTA1)/NuRD and the Cullin 4B (CUL4B)-Ring E3 ligase (CRL4B) complex to form a ternary complex. This complex catalyzed histone deacetylation and ubiquitination, inhibited the transcriptional activity of target genes, including PPARα, and promoted the proliferation and invasion of breast cancer cells in vitro. These physiological processes finally led to breast cancer occurrence, bone metastasis, and tumor stemness in vivo (Table 2) [110]. The above findings indicate that PPARα plays a role as a tumor suppressor in breast cancer.

PPARα was generally highly expressed in human primary inflammatory breast cancer cells SUM149PT (3.9-fold higher than primary human breast epithelial cells HMEC) and highly invasive breast cancer cells SUM1315MO2 (3.7-fold higher than HMEC cells) and in human breast tumor tissue (2–6-fold higher than adjacent normal tissues) [109]. The correlation between PPARα and breast cancer is worth further investigation.

### 4.2. PPARβ/δ and Breast Cancer

Among the three subtypes of PPARs, PPARβ/δ exhibits higher evolutionary efficiency [4]. In addition, uncoordinated PPARβ/δ also showed more potent transcriptional repression activity. Compared with uncoordinated PPARβ/δ, unligated PPARα and PPARγ do not inhibit PPRE-mediated transcription, which is possibly due to their inability to bind to the nuclear receptor corepressors such as SMRT and NCoR [111]. This relatively rapid rate of evolution and more potent transcriptional repression activity underscore the importance of investigating PPARβ/δ function. The PPARβ/δ are referred to as HUC-1 in humans [112], fatty acid-activated receptors (FAAR) in mice [113], and PPARδ in rats [114]. The PPARβ/δ are widely expressed in most tissues, and their expression level is often higher than that of PPARα and PPARγ. This widespread expression proves its importance in systemic activities and basic cell functions [52,115]. The high baseline expression of PPARγ, especially in the gastrointestinal tract and skeletal muscle, reveals the critical role of PPARβ/δ in fatty acid oxidation and obesity prevention [116]. PPARβ/δ is specific and diversified in cell fate. It can activate housekeeping genes and regulate energy metabolism. In addition, the endogenous natural ligands of PPARβ/δ are very broad and non-specific. The ability of these ligands to activate PPARβ/δ is relatively weak. Therefore, the physiological function of PPARβ/δ is difficult to simplify. Without ligand binding, PPARβ/δ degrades fast, while ligand binding inhibits ubiquitin-proteasome activity, thereby extending its half-life [117,118]. This phenomenon may also be attributed to ligand-induced PPARβ/δ expression [119]. Ligand-activated PPARβ/δ could increase the levels of serum high-density lipoprotein cholesterol, decrease the levels of serum triglycerides in mice [60], non-human primates [62], and humans [120], and improve the metabolic syndrome such as obesity and insulin resistance induced by a high-fat diet or genetic predisposition [116,121]. Inhibition of insulin resistance by activated PPARβ/δ might also improve progressive neurodegeneration and its associated learning and memory deficits and prevent Alzheimer’s disease [122,123]. In addition, PPARβ/δ also have considerable preventive or therapeutic capacity against genetic [124], diet [125], or chemically induced [126] liver inflammation.

The above evidence supports the development of PPARβ/δ specific agonists acting as clinical drugs for the treatment of diseases such as obesity, diabetes, metabolic syndrome, and liver inflammation. However, the synthesis of PPARβ/δ-targeted drugs has encountered significant obstacles related to clinical safety due to substantial controversy regarding the reports on the role of PPARβ/δ in cancer [127,128]. Ligand-activated PPARβ/δ could promote terminal differentiation of keratinocytes [129], enhance lipid deposition [130], inhibit cell proliferation [131], and inhibit the progression of skin cancers such as psoriasis. However, it has also been shown that transgenic mice that induced activation of PPARβ/δ in the epidermis developed an inflammatory skin disease strikingly similar to psoriasis. These mice were characterized by hyperproliferation of keratinocytes, aggregation of dendritic cells, and endothelial cell activation. The gene dysregulation and activation of key transcriptional programs and Th17 subsets of T cells in transgenic mice were also highly similar to psoriasis [132]. In addition, PPARβ/δ activated by UV stimulation directly promoted the expression of oncogene Src and upregulated its kinase activity, enhanced the EGFR/ERK1/2 signaling pathway, and promoted epithelial-mesenchymal transition (EMT), which promotes keratinocyte differentiation and proliferation [133]. This result also reveals the cancer-promoting effect of PPARβ/δ on skin cancer. A possible and one-sided explanation for this contradiction was that activation of PPARβ/δ existed both in keratinocytes and adjacent fibroblasts. The PPARβ/δ in fibroblasts inhibited IL-1 signaling by directly upregulating the expression of secreted interleukin-1 receptor antagonist (sIL-1ra), thereby regulating keratinocyte proliferation [134]. In addition to skin cancer, the PPARβ/δ also have a controversial role in colorectal cancer [40,135,136].

Human genome PPARβ/δ is located at 6p21.2, an increased site for ER- and high-risk breast cancer [137], which reveals the correlation between PPARβ/δ and breast cancer. PPARβ/δ was highly expressed in the nucleus in human normal breast epithelial cells and weakly expressed or even absent in 92% of human breast lobular and ductal cancer cells [138,139,140]. The expression of PPARβ/δ in mouse malignant breast cancer cells C20 was also significantly lower than that in mouse keratinocytes (nearly 4-fold) and human normal mammary epithelial cells MCF10A (more than 2-fold) [141]. The patients’ survival rate with breast cancer and the expression of PPARβ/δ have a negative correlation [142]. In 2004, Stephen et al. reported for the first time that PPARβ/δ activated by specific ligand compound F or GW501,516 could promote the proliferation of ER+ breast cancer cells MCF7 and T47D. It could also promote in T47D cells vascular endothelial growth factor α (VEGFα) and its receptor FLT-1 and encourage the proliferation of human umbilical vein endothelial cells (HUVEG) in vitro. However, activated PPARβ/δ did not exert similar effects on ER- breast cancer cells MDA-MB-231 and BT-20, revealing that the pro-proliferative and pro-angiogenic effects of PPARβ/δ on breast cancer are dependent on ER [143]. Conversely, in 2008, Girroir et al. reported that PPARβ/δ was activated by specific ligands (GW0742 or GW501,516) and inhibited the growth of MCF7 cells [144]. In 2010, Foreman et al. reported that PPARβ/δ activated by the above two ligands also inhibited proliferation and clone formation and promoted apoptosis in mouse C20 cells [141]. Additionally, in 2014, Yao et al. reported that the overexpression of PPARβ/δ prevented the proliferation of breast cancer cells, MDA-MB-231 and MCF7, while the treatment of the agonist GW0742 further inhibited the proliferation of MCF7 cells without any effect on the MDA-MB-231 cells. The overexpression of PPARβ/δ inhibited the clone formation of these two cell lines, while further treatment with GW0742 inhibited the clone formation of MDA-MB-231 cells significantly more than that of MCF7 cells. However, the overexpression or ligand-activated of PPARβ/δ did not affect apoptosis in either of the two breast cancer cell lines. Further, the overexpression of PPARβ/δ could inhibit the growth of xenograft tumor in MDA-MB-231 cells better than in MCF7 cells, and treatment with GW0742 further inhibited the volume of mouse xenografts [145]. These findings, although inconsistent with Stephen’s report [143], also confirm that the effects of PPARβ/δ on ER+ and ER- breast cancer cells were different. However, by real-time analysis of cell doubling time, Palkar et al. found that neither GW0742-activated nor highly specific irreversible antagonist GSK3787 inhibited PPARβ/δ had effects on the proliferation of MCF7 cells, despite the fact that both of them had the converse effect on the mRNA level of PPARβ/δ target gene Angptl4 in vitro and in vivo [30]. Additionally, although these disparate results may be attributed to the concentration of ligands used, cell treatment time, cell proliferation assessment methods, etc., the exact function of PPARβ/δ on breast cancer cell apoptosis and proliferation remains unclarified so far. Several experiments are required to reach consensus.

Ghosh et al. obtained PPARβ/δ−/−COX-2-TG transgenic mice by crossbreeding and found that PPARβ/δ silencing antagonized cyclooxygenase-2 (COX-2)-induced mammary gland hyperplasia and tumorigenesis in mice and significantly inhibited the expression of breast epithelial cell proliferation-related genes (e.g., Ki-67, Cyclin D1, etc.), revealing that PPARβ/δ plays the role of tumor suppressor in the development of breast cancer [146]. However, Glazer’s team found that treatment with GW501,516 accelerated adenosquamous carcinoma and mammary squamous cell tumor formation in mice induced with medroxyprogesterone acetate (MPA) and 7,12 dimethylbenzene(a)anthracene (DMBA). The elevated levels of PPARβ/δ were accompanied by increased activation of 3-phosphoinositide-dependent protein kinase 1 (PDK1), revealing that PPARβ/δ plays a role in promoting breast cancer development through the PDK1 signaling pathway [147]. PDK1 is a vital governor of the AGC protein kinase family, including all isoforms of the AKT/PKB, S6K, and PCK families [148]. Therefore, Glazer’s team constructed MMTV-PDK1 transgenic mice and found that overexpression of PDK1 in mouse mammary epithelial cells up-regulated the levels of pT308AKT and pS9GSK3β, as well as PPARβ/δ. After induction with MPA and DMBA, GW501,516 treated wild-type and transgenic mice showed an increased formation rate of mammary tumors compared with untreated normal wild-type mice. Further, between the two types of mice, the transgenic mice showed more pronounced tumors. The GW501,516 treatment did not alter PDK1 protein levels. In addition, PDK1 overexpression also enhanced PPARβ/δ-induced energy metabolism. These results reveal that PPARβ/δ promotes breast cancer by enhancing energy metabolism, which is dependent on PDK1/AKT signaling [149]. In 2013, Glazer’s team directly constructed MMTV-PPARβ/δ transgenic mice by embryo prokaryotic injection and found that overexpression of PPARβ/δ induced breast tumorigenesis and activation of the AKT/mTOR signaling pathway. The total number of mice developed invasive breast cancer within 12 months, and GW501,516 treatment strongly accelerated the oncogenic process and increased breast tumor diversity. A hallmark characteristic of MMTV-PPARβ/δ mice is the development of ER+/PR+/HER2- mammary tumors, further revealing the correlation between PPARβ/δ and ER+ ductal breast cancer [150]. The above experiments in vivo also reflect the conflicting roles of PPARβ/δ in breast cancer development, which may be attributed to the singleness of the GW501,516 therapeutic dose (0.005% (*w*/*w*)). In addition, as a specific agonist of PPARβ/δ, GW501,516 preferentially activates PPARβ/δ in human PPARs with a 667–833-fold higher affinity than the other two subtypes. However, the affinity of GW501,516 in mice is only 33–62-fold higher than that of other subtypes [151]. Therefore, this increased mammary tumorigenesis in mice treated with a single dose of GW501,516 may not be simply attributable to the activation of PPARβ/δ. However, it is undeniable that the successful construction of many transgenic mouse models is of great significance in studying the correlation between PPARβ/δ and breast cancer.

Retinoic acid (RA) as a tumor suppressor exhibits potent anticancer activity mediated by the nuclear retinoic acid receptor (RAR). The intracellular lipid-binding protein cellular retinoic acid-binding protein II (CRABP-II) targets RA to the RAR, while another lipid-binding protein, fatty acid binding protein 5 (FABP5), could deliver it to the non-canonical RA receptor PPARβ /δ. The FABP5/CRABP-II ratio determines the partition of RA between the two receptors. Noy’s team constructed two breast cancer MMTV-neu transgenic mouse models expressing different FABP5/CRABP-II ratios in breast tissue. It was observed that transgenic mice with a high FABP5/CRABP-II ratio produced larger breast tumors. On the contrary, the reduction of this ratio resulted in the suppression of breast tumor growth and gene expression, including PDK1 and cell proliferation-related genes, through the transfer of RA signaling from PPARβ/δ to RAR. This study proposes a new mechanism by which PPARβ/δ promote breast cancer [152]. Additionally, the epidermal growth factor receptor (EGFR) as a tumor-promoting factor can promote breast cancer cell proliferation and induce breast tumorigenesis. Noy’s team also found that treatment of MCF7 cells with the EGFR ligand heregulin-β1 could directly upregulate the expression of FABP5 and PDK1. The results indicated that FABP5 and PPARβ/δ were the key mediators of EGFR’s ability to enhance cell proliferation, further confirming that PPARβ/δ acted as a tumor-promoting factor playing a role in breast cancer [153]. However, studies on human keratinocyte HaCaT found that FABP5 neither delivered RA to PPARβ/δ nor promoted anti-apoptotic activity by upregulating PDK1 levels. This phenomenon was also identified in HaCaT cells that stably overexpress PPARβ/δ [154]. The above results suggest that the cancer-promoting effect of RA-mediated PPARβ/δ may be specific to breast cancer [155]. Wang et al. found that PPARβ/δ could promote the survival of MCF7 cells under rough microenvironmental conditions by reducing oxidative stress and promoting AKT-mediated survival signaling [156]. The correlation between PPARβ/δ and PDK1 is currently controversial. Although the above studies have found that the expression levels of the two are correlated, there are also studies showing that PDK1 is not a target gene of PPARβ/δ [136,155,157]. In addition to the research around the effect of PPARβ/δ on the proliferation and apoptosis of breast cancer cells, scholars have found that PPARβ/δ also has an effect on the invasion and metastasis of breast cancer cells. Adhikary found that PPARβ/δ, specifically antagonized by ST247 and DG172, inhibited serum and transforming growth factor β (TGFβ)-induced invasion of MDA-MB-231 cells [158]. However, Wang uncovered that the PPARβ/δ expression levels in more metastatic breast cancer basal cell lines were significantly higher than those in luminal cells. Additionally, after the inoculation with MCF7 cells overexpressing PPARβ/δ, the breast tumor volume and lung metastasis of mice increased significantly (Table 3) [156]. In conclusion, the exact role of PPARβ/δ on breast cancer still requires more experimental studies.

### 4.3. PPARγ and Breast Cancer

PPARγ1 and PPARγ2 are two isoforms of PPARγ, that were found in mice. The PPARγ2 mRNA was the predominant PPAR isoform in mouse mammary tissues [159]. In humans and monkeys, in addition to PPARγ1 and PPARγ2, a third isoform of PPARγ4 was found. These isoforms are the transcripts of seven mRNA spliceosomes (PPARγ1, PPARγ2, PPARγ3, PPARγ4, PPARγ5, PPARγ6, and PPARγ7) from the different transcription start sites, which are transcribed through alternative splicing of exons in the 5’-terminal region (A1, A2, B, C, and D) [160]. The PPARγ1, PPARγ3, PPARγ5, and PPARγ7 mRNAs translate into the same protein, PPARγ1, while PPARγ2 mRNA translates into PPARγ2 protein, whereas PPARγ4 and PPARγ6 mRNAs translate into the same PPARγ4 protein. PPARγ1 is expressed in almost all tissues, with the highest level in white and brown adipose tissues. Under normal physiological conditions, the larger PPARγ2 isoform (with additional amino acids at the amino-terminal of PPARγ2, 30 in mice and 28 in humans) is only expressed in brown and white adipose tissue, whereas its expression in the liver and skeletal muscle is caused by excessive caloric intake or genetic obesity. PPARγ4 is under-researched and expressed in macrophages and adipose tissues [161,162,163]. PPARγ widely expressed in white and brown adipose tissues, the large intestine, and the spleen. However, PPARγ is also found in the liver, pancreas, and tissues of the immune system [164]. A considerable number of studies have confirmed that ligand-activated PPARγ could regulate fat distribution and glucose and lipid metabolism [165] and reduce the inflammatory response of cardiovascular cells, especially endothelial cells [166]. Its specific agonist is relatively effective in the treatment of hyperlipidemia, hyperglycemia, and cardiovascular disease [167]. The specific agonists of PPARγ, i.e., TZDs, are clinical drugs currently on the market as insulin sensitizers for the treatment of type 2 diabetes, targeting PPARγ to exert a hypoglycemic effect. The antidiabetic activity of TZDs was first discovered in the early 1980s [168,169,170,171]. PPARγ is also involved in neural differentiation during the formation of neural precursor cells [83]. Therefore, its specific agonists could also act as protective agents for neurons, inducing synaptic plasticity and neurite outgrowth, and improving the symptoms of some neurological diseases [172]. In addition to the above effects, a large number of reports also pointed out that ligand-activated PPARγ exerts anti-tumor effects by promoting cell apoptosis and preventing cell proliferation, regulating cell metastasis, and stimulating angiogenesis, thereby inhibiting the occurrence and development of tumors of the liver [173], bladder [174], lung [175,176], brain [177], thyroid [178], esophagus [179] and colorectum [180,181,182,183].

PPARγ also plays a role in breast cancer progression. In 1998, it was reported that TZD-activated PPARγ could induce terminal differentiation of malignant mammary epithelial cells in vitro [184]. However, in 1999, researchers found that ligand-activated PPARγ could prevent the development of experimental breast cancer in vivo. The report showed that GW7845 as an activator of PPARγ significantly inhibited nitrosomethylurea (NMU)-induced mammary tumor incidence, tumor number, and tumor weight in rats [79]. Subsequent reports of ligand-activated PPARγ inhibiting breast cancer development have experienced a rise. A 2001 study showed that TGZ inhibited DMBA-induced mammary tumor progression in rats, reduced malignancy incidence, and induced regression or stasis of total tumor volume [108]. A study in 2009 showed that the conjugated fatty acid α-eleostearic acid (α-ESA) could act as an agonist of PPARγ, upregulating the level of PPARγ mRNA in MCF7 cells, upregulating PPARγ’s DNA binding activity and transcriptional activity, and mediating PPARγ nuclear translocation, thereby reducing MCF7 cell viability and promoting tumor cell apoptosis. At the same time, α-ESA-induced high PPARγ expression was associated with an inhibitory effect on ERK1/2 MAPK phosphorylation activation. This suggests that pERK1/2 might play a negative regulatory role on PPARγ levels [185]. Bonofiglio’s team discovered an important pathway for PPARγ in human breast cancer cell growth, cycle arrest, and apoptosis. RGZ-activated PPARγ inhibits the PI3K/AKT pathway and induces the antiproliferative effect of MCF7 cells [186]. RGZ also increased the binding of PPARγ to the NF-κB sequence on the promoter sequence of p53, upregulated the expression level of p53 in MCF7, induced caspase 9 cleavage and DNA fragmentation, triggered the apoptotic pathway, stopped the growth, and promoted apoptosis of breast cancer cells [187]. Furthermore, in several breast cancer cell lines, RGZ activated the human Fas ligand (FasL) promoter in a PPARγ-dependent manner, increased the binding of PPARγ with Sp1 to the Sp1 sequence located within the FasL promoter, and positively regulated FasL expression [188]. FasL is a type II transmembrane protein expressed on the membrane surface of activated T lymphocytes and cancer cells. By binding to its receptor Fas [189,190], it activates the cascade of caspases and induces apoptosis [191]. These studies reveal a novel molecular mechanism by which PPARγ induces growth arrest and apoptosis in breast cancer cells. An in vivo study in 2011 showed that TZD-activated PPARγ inhibited MAPK/STAT3/AKT phosphorylation-mediated leptin signaling in MCF7 cells. On one hand, this effect led to the inhibition of MCF7 xenografts through the counteraction of the stimulatory effects of leptin on estrogen signaling. On the other hand, it inhibited leptin-induced cell-cell aggregation and tumor cell proliferation, exerting pro-apoptotic and anti-proliferative effects on breast cancer cell lines [192].

Almost all experimental studies on PPARγ ligands reflect the prevention effect of these ligands on the occurrence and development of breast cancer. However, a 20-week human clinical trial found that the clinical value of TGZ treatment in patients with refractory metastatic breast cancer was not significant. All 22 patients receiving treatment displayed different levels of disease progression within 6 months. Some might even have started other systemic therapies. All patients with serum tumor marker expression above baseline had increased levels of these markers again within 8 weeks [193]. The public has been warned against TGZ by the U.S. Food and Drug Administration, and it was taken off the market in 2000 because of its specific hepatotoxicity [194]. It was subsequently withdrawn in the UK as well. In 1999 and 2000, RGZ and PGZ were marketed as targeted type 2 diabetes treatments in the US and Europe [195]. BGZ completed phase III clinical trials in 2010 and has not yet been listed [196]. However, short-term treatment with RGZ (2–6 weeks, *n* = 38) also did not protect tumor cell proliferation significantly in patients with an early stage of breast cancer [197]. Therefore, it is necessary to either synthesize new PPARγ activators with clinical value and few toxic side effects or find other drugs that can be used in combination with existing ligands for breast cancer treatment. In fact, as early as 1998, a study found that the combination of TGZ and all-trans-retinoic acid (ATRA) had a synergistic and irreversible inhibitory effect on the growth of MCF7 cells in vitro, induced MCF7 cell apoptosis, and was accompanied by a significant reduction of bcl-2. In vivo injection of the combined drug had no obvious toxic effects in mice. A drug combination could also significantly induce apoptosis and fibrosis-related morphological changes in breast cancer cells [198]. A 2008 study found that the PPARγ ligand N-(9-fluorenyl-methyloxycarbonyl)-l-leucine (F-L-Leu) combined with the COX-2 inhibitor celecoxib significantly delayed the median age of death in breast cancer mice. Breast cancer cell growth is also synergistically inhibited in vitro [199]. Bonofiglio’s team found that combining RGZ and RXR ligand 9-cis-retinoic acid (9RA) at nanomolar levels significantly inhibited the activity of breast cancer cells and promoted endogenous apoptosis. Combined treatment with RGZ and 9RA up-regulated the mRNA and protein levels of p53 and its effector gene p21 (WAF1/Cip1) in MCF7 cells, which led to a series of programmed apoptosis events such as the disruption of mitochondrial membrane potential, the release of cytochrome c, the activation of caspase 9, and DNA fragmentation [200]. The combination of CGZ and 9RA, another compound of the TZD family, could also synergistically prevent the human colon cancer cells’ Caco2 growth and induce apoptosis [201]. A 2011 study showed that the combination of TZD and the demethylating drug hydralazine could upregulate PPARγ transcriptional and translational levels in triple-negative breast cancer (TNBC) cells, thereby promoting the anti-proliferative and apoptotic effects of TNBC cells and reducing the xenograft tumor growth proliferation index [202]. In conclusion, the multi-drug combination regimen using PPARγ ligands could have a key role in the treatment of many malignant tumors, including breast cancer [203], ovarian cancer [204,205], colon cancer [206,207], and lung cancer [208,209].

In addition to its ligand-activated state, PPARγ also involves itself in the development of breast cancer in a non-ligand-independent manner. The PPARs and ERα are both members of the nuclear receptor superfamily. The ERα signaling pathway has a critical role in metabolism regulation and various physiological processes in the development of breast cancer [210,211]. Bonofiglio’s team found for the first time that ERα could bind to the PPRE element to inhibit its mediated transcriptional activity independently of PPARs. Interestingly, PPAR/RXR heterodimers could also bind to the ER response element (ERE) independently of ERs [212]. PPARγ physically interacted with ERα to form a ternary complex with a regulatory subunit of PI3K and p85. PPARγ and ERα played opposite roles in the regulation of PI3K/AKT signaling, which involves cell survival and proliferation [186]. The crosstalk between the PPARγ and ERα signaling pathways revealed the important role of PPARγ in the development of ER+ breast cancer. Since PPARγ-null mice are embryonic lethal, scientists have developed other ways to create transgenic animal models that silence PPARγ. Yin et al. investigated the susceptibility of PPARγ inactivation to MPA- and DMBA-induced breast cancer in mice by constructing an MMTV-Pax8PPARγ transgenic mouse model. In the absence of induction, the mammary glands of transgenic and wild-type mice did not differ in functional development or propensity for tumor formation, a finding consistent with Cui et al.’s [213]. However, after being induced by MPA and DMBA, transgenic mice developed higher tumor diversity than wild-type mice. These tumors were predominantly ER+ ductal breast cancers, further revealing the role of PPARγ in the development of ER+ breast cancer. The decrease in PTEN expression, the induction of pERK1 and pAKT levels, and decreasing pGSK3β level, Pax8PPARγ promotes Wnt signaling [214]. However, in constructing transgenic mice with constitutively active forms of MMTV-VpPPARγ, Saez et al. found that activation of PPARγ signaling did not affect mammary gland development in transgenic mice, which had no phenotypic difference with wild-type mice. On the other hand, when such transgenic mice were crossed with breast cancer-prone transgenic MMTV-PyV mice, the progeny biogenic mice developed tumors much faster and with a higher degree of malignancy and differentiation of the tumors. This molecular mechanism for promoting breast cancer development might also be attributed to the promotion of PPARγ on the Wnt signaling pathway [215]. Tian et al. conducted a parallel experiment on immunocompetent FVB mice, with one group of implanted tumor cells transduced with wild-type PPARγ, and the other with constitutively active PPARγCA. They found that the growth of mammary tumors in mice implanted with PPARγCA-transduced cells was enhanced, which was correlated with endothelial stem cells and angiogenesis increasing. PPARγCA induced ErbB2-transformed mammary epithelial cells to secrete Angptl4 protein, which enhanced angiogenesis in vivo and promoted tumor growth [216]. The above studies based on animal models reveal the contradictory roles (either inhibiting or promoting) of PPARγ in the occurrence and development of breast cancer. The potential reasons for this discrepancy remain to be investigated. The possible causes could be traced to the differences in the construction of animal models or the difference in the length of experimental periods. In addition, a 2019 study showed that PPARγ directly bound to the PPRE element of the protein tyrosine phosphatase receptor-type F (PTPRF) promoter and recruited RNA polymerase II and H3K4me3 to promote the transcription of PTPRF. These processes inhibited breast cancer cell proliferation and migration in vitro and inhibited breast tumor growth and distant metastasis in mice [217]. A 2020 experiment in vitro showed that PPARγ, which is commonly expressed in human primary and metastatic breast cancer [218], interacted with Nur77, recruited the ubiquitin E3 enzyme Trim13 to target the ubiquitin proteasomal degradation of Nur77, and promoted breast cancer progression. Nur77, a tumor suppressor, inhibits breast cancer cells from uptaking exogenous fatty acids and blocks the accumulation of fatty acids in the tumor metabolic microenvironment by inhibiting the transcription of the transmembrane protein CD36 and the cytoplasmic fatty acid-binding protein FABP4. Therefore, blocking the interaction between PPARγ and Nur77 can be used as a clinical approach for PPARγ ligand-independent treatment of breast cancer (Table 4) [219]. However, due to the relatively high concentrations of endogenous natural ligands in cells, it remains to be verified whether these conclusions are truly ligand-independent of PPARγ.

In 2005, an immunohistochemical test of 170 patients with invasive breast cancer showed that the expression of PPARγ was negatively associated with histological grade (*p* = 0.019). PPARγ had a significantly favorable effect on recurrence-free survival in breast ductal carcinoma patients (*p* = 0.027) and was an independent prognostic factor in ductal carcinoma patients (*p* = 0.039) [220]. In 2008, a study presented that the nuclear expression of PPARγ had a preventive effect on the recurrence of female breast ductal carcinoma in situ. Its expression level was negatively correlated with tumor recurrence (*p* = 0.024) [221]. These clinical research studies and the above experimental results reveal the important function of PPARγ in the occurrence and development of breast cancer. The overexpression of PPARγ in breast tumors and the physiological effects of its ligands on breast cancer cells indicate that PPARγ will be a possible target in breast cancer clinical prevention and treatment.

### 4.4. PPARs and TNBC

TNBC, the most aggressive subtype of breast cancer, has no effect on hormone therapy or HER2-targeted therapy due to its lack of the three receptors. Surgery or chemotherapy, the only viable option, is a systemic therapy that causes not only physical distress but a poor prognosis for TNBC patients [222]. Therefore, it is very necessary to explore new treatment methods or target drugs to improve the prognosis of TNBC. Li et al. found that the PPARα-specific agonist fenofibrate had anti-proliferative effects on breast cancer cell lines, and the top 5 most sensitive cells are all TNBC cell lines [223]. Kwong found that fatty acid binding protein 7 (FABP7) failed to induce the efficient use of glucose to generate ATP in the TNBC cell line Hs578T during serum starvation, eventually leading to cell death. This metabolic effect of FABP7 on Hs578T cells was mediated by PPARα [224]. Studies by Stephen’s group showed that PPARβ/δ activated by GW501,516 could promote the proliferation of MCF7 and T47D cells, but it had no similar effect on the TNBC cell lines MDA-MB-231 and BT-20 [143]. The expression level of PPARβ/δ in highly aggressive basal cells was significantly higher than that in luminal cells [156]. In addition, Adhikary’s team found that ST247 and DG172 specifically antagonized PPARβ/δ strongly inhibited the invasion ability of MDA-MB-231 cells induced by serum and TGFβ [158]. Jiang’s team found that the expression of PPARγ in the breast tissues of TNBC patients was significantly lower than that of other subtype patients, and its expression in MDA-MB-231 cells was also significantly lower than that of other breast cancer cell lines. Previous studies have reported that the PPARγ-specific agonist RGZ had antitumor effects in breast cancer. However, it did not exert significant anti-proliferative effects on MDA-MB-231 cells. RGZ combined with the demethylation agent hydralazine significantly inhibited the proliferation of MDA-MB-231 cells and promoted cell apoptosis [200]. Apaya et al. showed that epoxy-eicosatrienoic acid (EET) induced the nuclear translocation of FABP4 and FABP5 in MDA-MB-231 cells, thereby promoting the nuclear accumulation of PPARγ and affecting cell proliferation and migration [225]. These results reveal the important roles of all three subtypes of PPARs and their ligands in TNBC and suggest that more attention should be directed to drug combination therapies against TNBC.

## 5. Discussion

**Figure 6:**
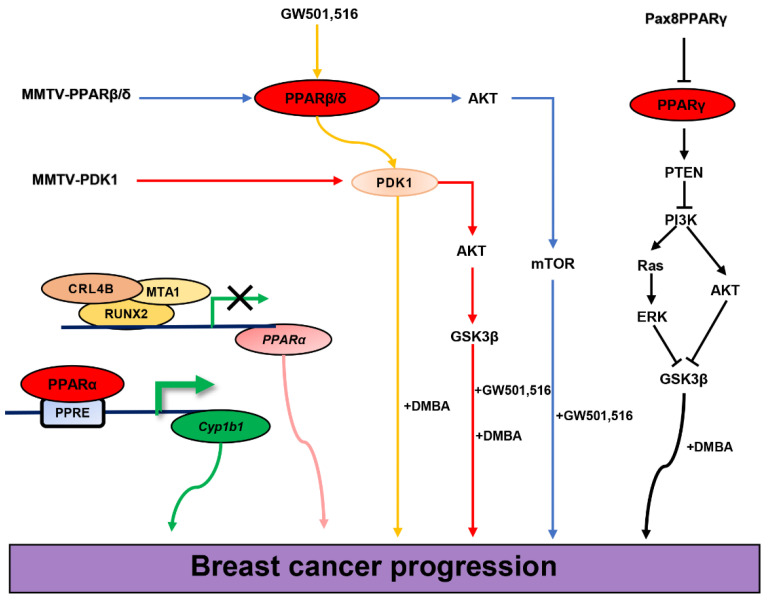
Schematic illustration of ligand-activated or ligand-independent PPARs affecting breast cancer progression. PPRE: peroxisome proliferator response element; *Cyp1b1*: cytochrome P450 1B1; RUNX2: Runt-related transcription factor 2; MTA1: metastasis-associated 1; CRL4B: Cullin 4B-Ring E3 ligase; PDK1: 3-phosphoinositide-dependent protein kinase 1; PTEN: phosphatase and tensin homolog; AKT: AKT serine/threonine kinase 1; GSK3β: glycogen synthase kinase 3β; mTOR: mechanistic target of rapamycin kinase; PI3K: phosphatidylinositol-4,5-bisphosphate 3-kinase; ERK: mitogen-activated protein kinase 1; DMBA: 7,12 dimethylbenzene(a)anthracene.

PPARs are key transcription factors in the process of fatty acid oxidative decomposition. They have a key role in nutrient metabolism and lipid homeostasis. The PPARs are involved in regulating several cellular physiological functions, consisting of cell differentiation, proliferation, metabolism, apoptosis, and other activities related to tumor formation. Several controversial reports on PPARs presented in this paper suggest that their function as tumor-promoting or tumor-suppressing factors in breast cancer still remains unclear. A number of classical signaling pathways in cells as a whole affect physiological function, such as cell carcinogenesis. The complexity of the pathways regulated by PPARs provides a one-sided explanation for their different functions in breast cancer (Figure 6). For example, both silence and constitutive activation of PPARγ enhanced Wnt signaling and promoted mammary tumorigenesis in transgenic mice [214,215]. GW501,516-activated PPARβ/δ promoted increased PDK1 activation in DMBA-induced mice [147]. The overexpression of PDK1 in mouse mammary epithelial cells in turn upregulated PPARβ/δ levels and enhanced PPARβ/δ-induced energy metabolism. However, GW501,516 treatment did not alter PDK1 protein levels [149]. Although the promoting effect of PPARβ/δ on breast cancer is partially dependent on the PDK1 signaling pathway, studies showed that PDK1 is not a target gene of PPARβ/δ [136,155,157], which further reveals the correlation between the two may be mediated by some factors in other signaling pathways. Many clinical drugs targeting PPARs (such as fibrate hypolipidemic drugs and TZD hypoglycemic drugs) can treat metabolic syndromes such as diabetes, obesity, hyperlipidemia, and cardiovascular disease. Moreover, epidemiological studies have shown that metabolic disorders are often associated with the occurrence of malignant tumors, such as breast cancer [226,227]. Therefore, PPARs remain a potential target for the prevention and treatment of breast cancer.

There are many predisposing factors for breast cancer, among which long-term estrogen exposure has been confirmed to be directly associated with the malignant proliferation, invasion, and metastasis of breast cancer cells [228]. ERs are the key factors in response to estrogen stimulation and mediate signal transduction and function in cells. Additionally, together with PPARs, they are members of the nuclear receptor superfamily. This review examined numerous reports on PPARs and found that regardless of the subtypes, the effects on ER+ and ER- breast cancer cells were different. Activated PPARα had the most significant pro-proliferation effect on ER+ MCF7 cells [104].Although the effect of PPARβ/δ on the proliferation of breast cancer cells is highly controversial, its effect on ER+ and ER- cells is indeed different [143,145]. A hallmark feature of MMTV-PPARβ/δ transgenic mice constructed by embryonic pronuclear injection developed ER+/PR+/HER2- mammary tumors, directly revealing the correlation between PPARβ/δ and ER+ ductal breast cancer [150]. PPARγ and ERα physically interacted to regulate the PI3K/AKT signaling pathway, which is involved in breast cancer cell survival and proliferation [186]. Further, MMTV-Pax8PPARγ transgenic mice produce mainly ER+ ductal breast cancer under the induction of MPA and DMBA [214]. This correlation between PPARs and ERs suggests that they can be used as synergistic targets for breast cancer clinical treatment. Consequently, the molecules and signals involved in regulating estrogen and its receptor pathways are very complex. They exhibit dynamic changes with differences in the intracellular environment. The function of PPARs in breast cancer is also disputable. Therefore, more experiments are needed for the development of common target drugs in the future.

The selectivity and affinity of various ligands for PPARs are different between humans and other mammals. This difference might be one of the causes of the opposite results obtained from experiments in vitro and in vivo. For example, Wy-14,643, an agonist of PPARα, enhanced the transcriptional activity of the tumor-promoting factor CYP1B1 in human MCF7 cells in vitro [106]. In turn, treatment with Wy-14,643 inhibited the ability of DMBA to induce mammary tumor formation in rats [108]. The GW501,516, an agonist of PPARβ/δ, induced the proliferation of human MCF7 and T47D cells [143]. However, it inhibited the proliferation and clone formation of mouse C20 cells and promoted cell apoptosis [141]. In addition to the interspecies specificity of ligands, the presence or absence of regulatory factors such as other native natural ligands in cells or mammals may also contribute to these conflicting results [151]. In addition to acting on its specific receptors, the fact that ligands have an effect on other substances is worth investigating. In addition, the compensatory effects of living organisms and cells, ligand-related pharmacokinetic behaviors, and weak activation or antagonism of high concentrations of ligands on other subtypes are all important factors that should be considered for inclusion or exclusion in future experiments [229].

PPARα has high expression in human breast cancer cells and tissues [104,109]. The PPARβ/δ is weakly expressed or absent in human breast lobular carcinoma and ductal carcinoma [138,139,140], and its expression level has a negative correlation with the survival rate of breast cancer patients [142]. PPARγ is generally highly expressed in human primary and metastatic breast cancer [218]. The expression of PPARγ is inversely correlated with the histological grade of invasive breast cancer [220] and with in situ ductal breast cancer recurrence [221]. It is an independent prognostic factor in patients with ductal carcinoma. This correlation revealed that PPARs would be potential clinical targets to prevent and treat breast cancer.

## 6. Conclusions

This review analyzed the roles and potential molecular mechanisms of three subtypes of PPARs in the presence or absence of ligands in breast cancer progression. In addition, the correlations between PPARs and ERs as the nuclear receptor superfamily and the investigation of the interaction between PPARs and key regulators in several signaling pathways were discussed. Furthermore, PPARs as targets for breast cancer prevention and treatment in order to provide more evidence for the synthesis of new drugs targeting PPARs or the search for new drug combination treatments. On the basis of the controversial results discovered in the review, further investigation is essential to reveal the physiological functions of PPARs.

## Figures and Tables

**Table 2:** The effects of PPARα on breast cancer.

	The Role in Breast Cancer	Binding Ligand	The Effect on Breast Cancer
PPARα	cancer-promoting	arachidonic acid	Promoted cell growth and proliferation, especially MCF7 in cells (ER++++) [104]
		Wy-14,643	Increased target gene CYP1B1 mRNA and protein levels in MCF7 cells promoted cancer progression [106]
		GW6471	Reduced cell viability, cell proliferation, and spheroid formation lead to apoptosis and metabolic dysfunction of stem cells [107]
	cancer-suppressing	Wy-14,643	Inhibited the ability of DMBA to induce tumor formation in rats and induced tumor volume regression [108]
		clofibrate	Inhibited cell proliferation and growth, affecting various lipid metabolism pathways [109]
		--	Inhibited the proliferation and invasion of cells in vitro, inhibited cancer occurrence, bone metastasis, and tumor stemness in vivo [110]

**Table 3:** The effects of PPARβ/δ on breast cancer.

	The Role in Breast Cancer	Binding Ligand	The Effect on Breast Cancer
PPARβ/δ	cancer-promoting	GW501,516	Promoted the proliferation of MCF7 and T47D cells (ER+) instead of MAD-MB-231 and BT-20 cells (ER-), promoted VEGFα and FLT-1 expression [143]
		GW501,516	Accelerated adenosquamous carcinoma and mammary squamous cell tumor formation in mice, increased activation of PDK1 [147]
		GW501,516	Accelerated tumor formation, did not alter PDK1 protein levels [149]
		GW501,516	Accelerated the oncogenic process and increased tumor diversity, especially ER+/PR+/HER2- tumors [150]
		--	Promoted tumor growth and the expression of genes, including PDK1 and cell proliferation-related genes [152]
		--	Promoted the expression of FABP5 and PDK1 in MCF7 cells, promoted cell proliferation, and induced tumorigenesis [153]
		--	Promoted the survival of MCF7 cells under harsh microenvironmental conditions [156]
		ST247 or DG172	Inhibited serum and TGFβ-induced invasion of MDA-MB-231 cells [158]
		--	Increased tumor volume and lung metastasis in mice [156]
	cancer-suppressing	GW0742 or GW501,516	Inhibited the growth of MCF7 cells [144]
		GW0742 or GW501,516	Inhibited the proliferation and clone formation, and promoted apoptosis in mouse C20 cells [141]
		GW0742	Inhibited the proliferation of MCF7 cells instead of MDA-MB-231 cells, inhibited the clone formation of MDA-MB-231 cells significantly more than that of MCF7 cells, and inhibited the volume of mouse xenografts [145]
		--	Inhibited hyperplasia and tumorigenesis in mice and inhibited the expression of epithelial cell proliferation-related genes (e.g., Ki-67, Cyclin D1, etc.) [146]
	no effect	GW0742 or GSK3787	Had no effect on the proliferation of MCF7 cells, despite both of them influencing the mRNA level of the target gene Angptl4 in vitro and in vivo [30]

**Table 4:** The effects of PPARγ on breast cancer.

	The Role in Breast Cancer	Binding Ligand	The Effect on Breast Cancer
PPARγ	cancer-promoting	--	Promoted Wnt signaling and induced transgenic mice to develop tumors much faster with a higher degree of malignancy and differentiation of the tumors [215]
		--	Promoted the growth of tumors and angiogenesis in mice, increasing Angptl4 expression and endothelial stem cells [216]
		--	Interacted with Nur77, recruited Trim13 to target the ubiquitin proteasomal degradation of Nur77, and promoted cancer progression [219]
	cancer-suppressing	TZD	Induced terminal differentiation of malignant mammary epithelial cells [184]
		GW7845	Inhibited NMU-induced tumor incidence, tumor number, and tumor weight in rats [79].
		TGZ	Inhibited DMBA-induced tumor progression in rats, reduced malignancy incidence, and induced regression or stasis of total tumor volume [108]
		α-eleostearic acid	Reduced MCF7 cell viability and promoted cell apoptosis [185]
		RGZ	Inhibited PI3K/AKT pathway, inhibited proliferation of MCF7 cells [186]
		RGZ	Promoted the expression of p53 in MCF7, induced caspase 9 cleavage and DNA fragmentation, and promoted cell growth arrest and apoptosis [187]
		RGZ	Promoted target gene FasL expression, activated the cascade of caspases, and induced apoptosis [191]
		TZD	Inhibited MAPK/STAT3/AKT phosphorylation-mediated leptin signaling in MCF7 cells inhibited cell proliferation and promoted cell apoptosis [192]
		BRL49,653	Inhibited the PI3K/AKT pathway and promoted PTEN expression in MCF7 cells, inhibiting cell growth [186]
		--	PPARγ silcence promoted Wnt signaling and induced transgenic mice to develope higher tumor diversity, especially ER+ ductal tumors [214]
		--	Inhibited cell proliferation and migration in vitro, inhibited tumor growth, and distant metastasis in mice [217]

## Data Availability

Not applicable.

## References

[1] Siegel R.L., Miller K.D., Fuchs H.E., Jemal A. (2022). Cancer statistics, 2022. CA Cancer J. Clin..

[2] Perou C.M., Sørlie T., Eisen M.B., van de Rijn M., Jeffrey S.S., Rees C.A., Pollack J.R., Ross D.T., Johnsen H., Akslen L.A. (2000). Molecular portraits of human breast tumours. Nature.

[3] Sorlie T., Tibshirani R., Parker J., Hastie T., Marron J.S., Nobel A., Deng S., Johnsen H., Pesich R., Geisler S. (2003). Repeated observation of breast tumor subtypes in independent gene expression data sets. Proc. Natl. Acad. Sci. USA.

[4] Issemann I., Green S. (1990). Activation of a member of the steroid hormone receptor superfamily by peroxisome proliferators. Nature.

[5] Escher P., Wahli W. (2000). Peroxisome proliferator-activated receptors: Insight into multiple cellular functions. Mutat. Res..

[6] Feige J.N., Gelman L., Michalik L., Desvergne B., Wahli W. (2006). From molecular action to physiological outputs: Peroxisome proliferator-activated receptors are nuclear receptors at the crossroads of key cellular functions. Prog. Lipid Res..

[7] Polvani S., Tarocchi M., Tempesti S., Bencini L., Galli A. (2016). Peroxisome proliferator activated receptors at the crossroad of obesity, diabetes, and pancreatic cancer. World J. Gastroenterol..

[8] Wang Y.-X. (2010). PPARs: Diverse regulators in energy metabolism and metabolic diseases. Cell Res..

[9] Han L., Shen W.-J., Bittner S., Kraemer F.B., Azhar S. (2017). PPARs: Regulators of metabolism and as therapeutic targets in cardiovascular disease. Part I: PPAR-α. Future Cardiol..

[10] Han L., Shen W.-J., Bittner S., Kraemer F.B., Azhar S. (2017). PPARs: Regulators of metabolism and as therapeutic targets in cardiovascular disease. Part II: PPAR-β/δ and PPAR-γ. Future Cardiol..

[11] Burns K.A., Vanden Heuvel J.P. (2007). Modulation of PPAR activity via phosphorylation. Biochim. Biophys. Acta.

[12] Castillo G., Brun R.P., Rosenfield J.K., Hauser S., Park C.W., Troy A.E., Wright M.E., Spiegelman B.M. (1999). An adipogenic cofactor bound by the differentiation domain of PPARgamma. EMBO J..

[13] Hummasti S., Tontonoz P. (2006). The peroxisome proliferator-activated receptor N-terminal domain controls isotype-selective gene expression and adipogenesis. Mol. Endocrinol..

[14] Bugge A., Grøntved L., Aagaard M.M., Borup R., Mandrup S. (2009). The PPARgamma2 A/B-domain plays a gene-specific role in transactivation and cofactor recruitment. Mol. Endocrinol..

[15] Gampe R.T.J., Montana V.G., Lambert M.H., Miller A.B., Bledsoe R.K., Milburn M.V., Kliewer S.A., Willson T.M., Xu H.E. (2000). Asymmetry in the PPARgamma/RXRalpha crystal structure reveals the molecular basis of heterodimerization among nuclear receptors. Mol. Cell.

[16] Nolte R.T., Wisely G.B., Westin S., Cobb J.E., Lambert M.H., Kurokawa R., Rosenfeld M.G., Willson T.M., Glass C.K., Milburn M.V. (1998). Ligand binding and co-activator assembly of the peroxisome proliferator-activated receptor-gamma. Nature.

[17] Sheu S.-H., Kaya T., Waxman D.J., Vajda S. (2005). Exploring the binding site structure of the PPAR gamma ligand-binding domain by computational solvent mapping. Biochemistry.

[18] Xu H.E., Lambert M.H., Montana V.G., Parks D.J., Blanchard S.G., Brown P.J., Sternbach D.D., Lehmann J.M., Wisely G.B., Willson T.M. (1999). Molecular recognition of fatty acids by peroxisome proliferator-activated receptors. Mol. Cell.

[19] Shao D., Rangwala S.M., Bailey S.T., Krakow S.L., Reginato M.J., Lazar M.A. (1998). Interdomain communication regulating ligand binding by PPAR-gamma. Nature.

[20] Deeb S.S., Fajas L., Nemoto M., Pihlajamäki J., Mykkänen L., Kuusisto J., Laakso M., Fujimoto W., Auwerx J. (1998). A Pro12Ala substitution in PPARgamma2 associated with decreased receptor activity, lower body mass index and improved insulin sensitivity. Nat. Genet..

[21] Lee C.-H., Chawla A., Urbiztondo N., Liao D., Boisvert W.A., Evans R.M., Curtiss L.K. (2003). Transcriptional repression of atherogenic inflammation: Modulation by PPARdelta. Science.

[22] Hall J.M., McDonnell D.P. (2005). Coregulators in nuclear estrogen receptor action: From concept to therapeutic targeting. Mol. Interv..

[23] Göttlicher M., Widmark E., Li Q., Gustafsson J.A. (1992). Fatty acids activate a chimera of the clofibric acid-activated receptor and the glucocorticoid receptor. Proc. Natl. Acad. Sci. USA.

[24] Dreyer C., Krey G., Keller H., Givel F., Helftenbein G., Wahli W. (1992). Control of the peroxisomal beta-oxidation pathway by a novel family of nuclear hormone receptors. Cell.

[25] Ziouzenkova O., Perrey S., Asatryan L., Hwang J., MacNaul K.L., Moller D.E., Rader D.J., Sevanian A., Zechner R., Hoefler G. (2003). Lipolysis of triglyceride-rich lipoproteins generates PPAR ligands: Evidence for an antiinflammatory role for lipoprotein lipase. Proc. Natl. Acad. Sci. USA.

[26] Chawla A., Lee C.-H., Barak Y., He W., Rosenfeld J., Liao D., Han J., Kang H., Evans R.M. (2003). PPARdelta is a very low-density lipoprotein sensor in macrophages. Proc. Natl. Acad. Sci. USA.

[27] Yu K., Bayona W., Kallen C.B., Harding H.P., Ravera C.P., McMahon G., Brown M., Lazar M.A. (1995). Differential activation of peroxisome proliferator-activated receptors by eicosanoids. J. Biol. Chem..

[28] Willson T.M., Brown P.J., Sternbach D.D., Henke B.R. (2000). The PPARs: From orphan receptors to drug discovery. J. Med. Chem..

[29] Shearer B.G., Steger D.J., Way J.M., Stanley T.B., Lobe D.C., Grillot D.A., Iannone M.A., Lazar M.A., Willson T.M., Billin A.N. (2008). Identification and characterization of a selective peroxisome proliferator-activated receptor beta/delta (NR1C2) antagonist. Mol. Endocrinol..

[30] Palkar P.S., Borland M.G., Naruhn S., Ferry C.H., Lee C., Sk U.H., Sharma A.K., Amin S., Murray I.A., Anderson C.R. (2010). Cellular and pharmacological selectivity of the peroxisome proliferator-activated receptor-beta/delta antagonist GSK3787. Mol. Pharmacol..

[31] Dowell P., Peterson V.J., Zabriskie T.M., Leid M. (1997). Ligand-induced peroxisome proliferator-activated receptor alpha conformational change. J. Biol. Chem..

[32] Tien E.S., Hannon D.B., Thompson J.T., Vanden Heuvel J.P. (2006). Examination of Ligand-Dependent Coactivator Recruitment by Peroxisome Proliferator-Activated Receptor-alpha (PPARalpha). PPAR Res..

[33] Sumanasekera W.K., Tien E.S., Turpey R., Vanden Heuvel J.P., Perdew G.H. (2003). Evidence that peroxisome proliferator-activated receptor alpha is complexed with the 90-kDa heat shock protein and the hepatitis virus B X-associated protein 2. J. Biol. Chem..

[34] Sumanasekera W.K., Tien E.S., Davis J.W., Turpey R., Perdew G.H., Vanden Heuvel J.P. (2003). Heat shock protein-90 (Hsp90) acts as a repressor of peroxisome proliferator-activated receptor-alpha (PPARalpha) and PPARbeta activity. Biochemistry.

[35] Akiyama T.E., Baumann C.T., Sakai S., Hager G.L., Gonzalez F.J. (2002). Selective intranuclear redistribution of PPAR isoforms by RXR alpha. Mol. Endocrinol..

[36] Patel H., Truant R., Rachubinski R.A., Capone J.P. (2005). Activity and subcellular compartmentalization of peroxisome proliferator-activated receptor alpha are altered by the centrosome-associated protein CAP350. J. Cell Sci..

[37] Malek M.A., Hoang M.-H., Jia Y., Lee J.H., Jun H.J., Lee D.-H., Lee H.J., Lee C., Lee M.K., Hwang B.Y. (2013). Ombuin-3-O-β-D-glucopyranoside from Gynostemma pentaphyllum is a dual agonistic ligand of peroxisome proliferator-activated receptors α and δ/β. Biochem. Biophys. Res. Commun..

[38] Tenenbaum A., Motro M., Fisman E.Z. (2005). Dual and pan-peroxisome proliferator-activated receptors (PPAR) co-agonism: The bezafibrate lessons. Cardiovasc. Diabetol..

[39] Nagy L., Tontonoz P., Alvarez J.G., Chen H., Evans R.M. (1998). Oxidized LDL regulates macrophage gene expression through ligand activation of PPARgamma. Cell.

[40] Shureiqi I., Jiang W., Zuo X., Wu Y., Stimmel J.B., Leesnitzer L.M., Morris J.S., Fan H.-Z., Fischer S.M., Lippman S.M. (2003). The 15-lipoxygenase-1 product 13-S-hydroxyoctadecadienoic acid down-regulates PPAR-delta to induce apoptosis in colorectal cancer cells. Proc. Natl. Acad. Sci. USA.

[41] Coleman J.D., Prabhu K.S., Thompson J.T., Reddy P.S., Peters J.M., Peterson B.R., Reddy C.C., Vanden Heuvel J.P. (2007). The oxidative stress mediator 4-hydroxynonenal is an intracellular agonist of the nuclear receptor peroxisome proliferator-activated receptor-beta/delta (PPARbeta/delta). Free Radic. Biol. Med..

[42] Rubenstrunk A., Hanf R., Hum D.W., Fruchart J.-C., Staels B. (2007). Safety issues and prospects for future generations of PPAR modulators. Biochim. Biophys. Acta.

[43] Tenenbaum A., Boyko V., Fisman E.Z., Goldenberg I., Adler Y., Feinberg M.S., Motro M., Tanne D., Shemesh J., Schwammenthal E. (2008). Does the lipid-lowering peroxisome proliferator-activated receptors ligand bezafibrate prevent colon cancer in patients with coronary artery disease?. Cardiovasc. Diabetol..

[44] Yaturu S., Bryant B., Jain S.K. (2007). Thiazolidinedione treatment decreases bone mineral density in type 2 diabetic men. Diabetes Care.

[45] Grey A., Bolland M., Gamble G., Wattie D., Horne A., Davidson J., Reid I.R. (2007). The peroxisome proliferator-activated receptor-gamma agonist rosiglitazone decreases bone formation and bone mineral density in healthy postmenopausal women: A randomized, controlled trial. J. Clin. Endocrinol. Metab..

[46] Schwartz A.V., Sellmeyer D.E. (2007). Thiazolidinedione therapy gets complicated: Is bone loss the price of improved insulin resistance?. Diabetes Care.

[47] Schwartz A.V., Sellmeyer D.E., Vittinghoff E., Palermo L., Lecka-Czernik B., Feingold K.R., Strotmeyer E.S., Resnick H.E., Carbone L., Beamer B.A. (2006). Thiazolidinedione use and bone loss in older diabetic adults. J. Clin. Endocrinol. Metab..

[48] Still K., Grabowski P., Mackie I., Perry M., Bishop N. (2008). The peroxisome proliferator activator receptor alpha/delta agonists linoleic acid and bezafibrate upregulate osteoblast differentiation and induce periosteal bone formation in vivo. Calcif. Tissue Int..

[49] Rakhshandehroo M., Knoch B., Müller M., Kersten S. (2010). Peroxisome proliferator-activated receptor alpha target genes. PPAR Res..

[50] Santilli A.A., Scotese A.C., Tomarelli R.M. (1974). A potent antihypercholesterolemic agent: (4-chloro-6-(2,3-xylidino)-2-pyrimidinylthio) acetic acid (Wy-14643). Experientia.

[51] Reddy J.K., Moody D.E., Azarnoff D.L., Tomarelli R.M. (1977). Hepatic effects of some [4-chloro-6-(2,3-xylidino)-2-pyrimidinylthio] acetic acid (WY-14,643) analogs in the mouse. Arch. Int. Pharmacodyn. Ther..

[52] Kliewer S.A., Forman B.M., Blumberg B., Ong E.S., Borgmeyer U., Mangelsdorf D.J., Umesono K., Evans R.M. (1994). Differential expression and activation of a family of murine peroxisome proliferator-activated receptors. Proc. Natl. Acad. Sci. USA.

[53] Fan S., Gao Y., Qu A., Jiang Y., Li H., Xie G., Yao X., Yang X., Zhu S., Yagai T. (2022). YAP-TEAD mediates PPAR α-induced hepatomegaly and liver regeneration in mice. Hepatology.

[54] Brown P.J., Winegar D.A., Plunket K.D., Moore L.B., Lewis M.C., Wilson J.G., Sundseth S.S., Koble C.S., Wu Z., Chapman J.M. (1999). A ureido-thioisobutyric acid (GW9578) is a subtype-selective PPARalpha agonist with potent lipid-lowering activity. J. Med. Chem..

[55] Brown P.J., Stuart L.W., Hurley K.P., Lewis M.C., Winegar D.A., Wilson J.G., Wilkison W.O., Ittoop O.R., Willson T.M. (2001). Identification of a subtype selective human PPARα agonist through parallel-array synthesis. Bioorg. Med. Chem. Lett..

[56] Xu H.E., Stanley T.B., Montana V.G., Lambert M.H., Shearer B.G., Cobb J.E., McKee D.D., Galardi C.M., Plunket K.D., Nolte R.T. (2002). Structural basis for antagonist-mediated recruitment of nuclear co-repressors by PPARalpha. Nature.

[57] Guhlmann A., Keppler A., Kästner S., Krieter H., Brückner U.B., Messmer K., Keppler D. (1989). Prevention of endogenous leukotriene production during anaphylaxis in the guinea pig by an inhibitor of leukotriene biosynthesis (MK-886) but not by dexamethasone. J. Exp. Med..

[58] Datta K., Biswal S.S., Kehrer J.P. (1999). The 5-lipoxygenase-activating protein (FLAP) inhibitor, MK886, induces apoptosis independently of FLAP. Biochem. J..

[59] Kehrer J.P., Biswal S.S., La E., Thuillier P., Datta K., Fischer S.M., Vanden Heuvel J.P. (2001). Inhibition of peroxisome-proliferator-activated receptor (PPAR)alpha by MK886. Biochem. J..

[60] Leibowitz M.D., Fiévet C., Hennuyer N., Peinado-Onsurbe J., Duez H., Bergera J., Cullinan C.A., Sparrow C.P., Baffic J., Berger G.D. (2000). Activation of PPARdelta alters lipid metabolism in db/db mice. FEBS Lett..

[61] Westergaard M., Henningsen J., Svendsen M.L., Johansen C., Jensen U.B., Schrøder H.D., Kratchmarova I., Berge R.K., Iversen L., Bolund L. (2001). Modulation of keratinocyte gene expression and differentiation by PPAR-selective ligands and tetradecylthioacetic acid. J. Investig. Dermatol..

[62] Oliver W.R.J., Shenk J.L., Snaith M.R., Russell C.S., Plunket K.D., Bodkin N.L., Lewis M.C., Winegar D.A., Sznaidman M.L., Lambert M.H. (2001). A selective peroxisome proliferator-activated receptor delta agonist promotes reverse cholesterol transport. Proc. Natl. Acad. Sci. USA.

[63] Pelton P. (2006). GW-501516 GlaxoSmithKline/Ligand. Curr. Opin. Investig. Drugs.

[64] Iwaisako K., Haimerl M., Paik Y.-H., Taura K., Kodama Y., Sirlin C., Yu E., Yu R.T., Downes M., Evans R.M. (2012). Protection from liver fibrosis by a peroxisome proliferator-activated receptor δ agonist. Proc. Natl. Acad. Sci. USA.

[65] Lamers C., Schubert-Zsilavecz M., Merk D. (2012). Therapeutic modulators of peroxisome proliferator-activated receptors (PPAR): A patent review (2008-present). Expert Opin. Ther. Pat..

[66] Lin Y., Zhu X., McLntee F.L., Xiao H., Zhang J., Fu M., Chen Y.E. (2004). Interferon regulatory factor-1 mediates PPARgamma-induced apoptosis in vascular smooth muscle cells. Arterioscler. Thromb. Vasc. Biol..

[67] Choi Y.-J., Roberts B.K., Wang X., Geaney J.C., Naim S., Wojnoonski K., Karpf D.B., Krauss R.M. (2012). Effects of the PPAR-δ agonist MBX-8025 on atherogenic dyslipidemia. Atherosclerosis.

[68] Billin A.N. (2008). PPAR-beta/delta agonists for Type 2 diabetes and dyslipidemia: An adopted orphan still looking for a home. Expert Opin. Investig. Drugs.

[69] Seimandi M., Lemaire G., Pillon A., Perrin A., Carlavan I., Voegel J.J., Vignon F., Nicolas J.-C., Balaguer P. (2005). Differential responses of PPARalpha, PPARdelta, and PPARgamma reporter cell lines to selective PPAR synthetic ligands. Anal. Biochem..

[70] Zaveri N.T., Sato B.G., Jiang F., Calaoagan J., Laderoute K.R., Murphy B.J. (2009). A novel peroxisome proliferator-activated receptor delta antagonist, SR13904, has anti-proliferative activity in human cancer cells. Cancer Biol. Ther..

[71] Shearer B.G., Wiethe R.W., Ashe A., Billin A.N., Way J.M., Stanley T.B., Wagner C.D., Xu R.X., Leesnitzer L.M., Merrihew R.V. (2010). Identification and characterization of 4-chloro-N-(2-{[5-trifluoromethyl)-2-pyridyl]sulfonyl}ethyl)benzamide (GSK3787), a selective and irreversible peroxisome proliferator-activated receptor delta (PPARdelta) antagonist. J. Med. Chem..

[72] Lieber S., Scheer F., Meissner W., Naruhn S., Adhikary T., Müller-Brüsselbach S., Diederich W.E., Müller R. (2012). (Z)-2-(2-bromophenyl)-3-{[4-(1-methyl-piperazine)amino]phenyl}acrylonitrile (DG172): An orally bioavailable PPARβ/δ-selective ligand with inverse agonistic properties. J. Med. Chem..

[73] Levi L., Lobo G., Doud M.K., von Lintig J., Seachrist D., Tochtrop G.P., Noy N. (2013). Genetic ablation of the fatty acid-binding protein FABP5 suppresses HER2-induced mammary tumorigenesis. Cancer Res..

[74] Naruhn S., Toth P.M., Adhikary T., Kaddatz K., Pape V., Dörr S., Klebe G., Müller-Brüsselbach S., Diederich W.E., Müller R. (2011). High-affinity peroxisome proliferator-activated receptor β/δ-specific ligands with pure antagonistic or inverse agonistic properties. Mol. Pharmacol..

[75] Lehmann J.M., Moore L.B., Smith-Oliver T.A., Wilkison W.O., Willson T.M., Kliewer S.A. (1995). An antidiabetic thiazolidinedione is a high affinity ligand for peroxisome proliferator-activated receptor gamma (PPAR gamma). J. Biol. Chem..

[76] Mirza A.Z., Althagafi I.I., Shamshad H. (2019). Role of PPAR receptor in different diseases and their ligands: Physiological importance and clinical implications. Eur. J. Med. Chem..

[77] Elbrecht A., Chen Y., Adams A., Berger J., Griffin P., Klatt T., Zhang B., Menke J., Zhou G., Smith R.G. (1999). L-764406 is a partial agonist of human peroxisome proliferator-activated receptor gamma. The role of Cys313 in ligand binding. J. Biol. Chem..

[78] Oberfield J.L., Collins J.L., Holmes C.P., Goreham D.M., Cooper J.P., Cobb J.E., Lenhard J.M., Hull-Ryde E.A., Mohr C.P., Blanchard S.G. (1999). A peroxisome proliferator-activated receptor gamma ligand inhibits adipocyte differentiation. Proc. Natl. Acad. Sci. USA.

[79] Suh N., Wang Y., Williams C.R., Risingsong R., Gilmer T., Willson T.M., Sporn M.B. (1999). A new ligand for the peroxisome proliferator-activated receptor-gamma (PPAR-gamma), GW7845, inhibits rat mammary carcinogenesis. Cancer Res..

[80] Miyahara T., Schrum L., Rippe R., Xiong S., Yee H.F.J., Motomura K., Anania F.A., Willson T.M., Tsukamoto H. (2000). Peroxisome proliferator-activated receptors and hepatic stellate cell activation. J. Biol. Chem..

[81] Leesnitzer L.M., Parks D.J., Bledsoe R.K., Cobb J.E., Collins J.L., Consler T.G., Davis R.G., Hull-Ryde E.A., Lenhard J.M., Patel L. (2002). Functional consequences of cysteine modification in the ligand binding sites of peroxisome proliferator activated receptors by GW9662. Biochemistry.

[82] Ji J., Xue T.-F., Guo X.-D., Yang J., Guo R.-B., Wang J., Huang J.-Y., Zhao X.-J., Sun X.-L. (2018). Antagonizing peroxisome proliferator-activated receptor γ facilitates M1-to-M2 shift of microglia by enhancing autophagy via the LKB1-AMPK signaling pathway. Aging Cell.

[83] Ghoochani A., Shabani K., Peymani M., Ghaedi K., Karamali F., Karbalaei K., Tanhaie S., Salamian A., Esmaeili A., Valian-Borujeni S. (2012). The influence of peroxisome proliferator-activated receptor γ(1) during differentiation of mouse embryonic stem cells to neural cells. Differentiation.

[84] Wright H.M., Clish C.B., Mikami T., Hauser S., Yanagi K., Hiramatsu R., Serhan C.N., Spiegelman B.M. (2000). A synthetic antagonist for the peroxisome proliferator-activated receptor gamma inhibits adipocyte differentiation. J. Biol. Chem..

[85] Berger J., Moller D.E. (2002). The mechanisms of action of PPARs. Annu. Rev. Med..

[86] Fang L., Zhang M., Li Y., Liu Y., Cui Q., Wang N. (2016). PPARgene: A Database of Experimentally Verified and Computationally Predicted PPAR Target Genes. PPAR Res..

[87] Peters J.M., Foreman J.E., Gonzalez F.J. (2011). Dissecting the role of peroxisome proliferator-activated receptor-β/δ (PPARβ/δ) in colon, breast, and lung carcinogenesis. Cancer Metastasis Rev..

[88] Tontonoz P., Spiegelman B.M. (2008). Fat and beyond: The diverse biology of PPARgamma. Annu. Rev. Biochem..

[89] Escher P., Braissant O., Basu-Modak S., Michalik L., Wahli W., Desvergne B. (2001). Rat PPARs: Quantitative analysis in adult rat tissues and regulation in fasting and refeeding. Endocrinology.

[90] Pyper S.R., Viswakarma N., Yu S., Reddy J.K. (2010). PPARalpha: Energy combustion, hypolipidemia, inflammation and cancer. Nucl. Recept. Signal..

[91] Staels B., Dallongeville J., Auwerx J., Schoonjans K., Leitersdorf E., Fruchart J.C. (1998). Mechanism of action of fibrates on lipid and lipoprotein metabolism. Circulation.

[92] Balfour J.A., McTavish D., Heel R.C. (1990). Fenofibrate. A review of its pharmacodynamic and pharmacokinetic properties and therapeutic use in dyslipidaemia. Drugs.

[93] Reddy J.K., Azarnoff D.L., Hignite C.E. (1980). Hypolipidaemic hepatic peroxisome proliferators form a novel class of chemical carcinogens. Nature.

[94] Peters J.M., Cattley R.C., Gonzalez F.J. (1997). Role of PPAR alpha in the mechanism of action of the nongenotoxic carcinogen and peroxisome proliferator Wy-14,643. Carcinogenesis.

[95] Hays T., Rusyn I., Burns A.M., Kennett M.J., Ward J.M., Gonzalez F.J., Peters J.M. (2005). Role of peroxisome proliferator-activated receptor-alpha (PPARalpha) in bezafibrate-induced hepatocarcinogenesis and cholestasis. Carcinogenesis.

[96] Peters J.M., Cheung C., Gonzalez F.J. (2005). Peroxisome proliferator-activated receptor-alpha and liver cancer: Where do we stand?. J. Mol. Med..

[97] Shah Y.M., Morimura K., Yang Q., Tanabe T., Takagi M., Gonzalez F.J. (2007). Peroxisome proliferator-activated receptor alpha regulates a microRNA-mediated signaling cascade responsible for hepatocellular proliferation. Mol. Cell. Biol..

[98] Cheung C., Akiyama T.E., Ward J.M., Nicol C.J., Feigenbaum L., Vinson C., Gonzalez F.J. (2004). Diminished hepatocellular proliferation in mice humanized for the nuclear receptor peroxisome proliferator-activated receptor alpha. Cancer Res..

[99] Morimura K., Cheung C., Ward J.M., Reddy J.K., Gonzalez F.J. (2006). Differential susceptibility of mice humanized for peroxisome proliferator-activated receptor alpha to Wy-14,643-induced liver tumorigenesis. Carcinogenesis.

[100] Holland C.M., Saidi S.A., Evans A.L., Sharkey A.M., Latimer J.A., Crawford R.A.F., Charnock-Jones D.S., Print C.G., Smith S.K. (2004). Transcriptome analysis of endometrial cancer identifies peroxisome proliferator-activated receptors as potential therapeutic targets. Mol. Cancer Ther..

[101] Nickkho-Amiry M., McVey R., Holland C. (2012). Peroxisome proliferator-activated receptors modulate proliferation and angiogenesis in human endometrial carcinoma. Mol. Cancer Res..

[102] Jiao H., Zhao B. (2002). Cytotoxic effect of peroxisome proliferator fenofibrate on human HepG2 hepatoma cell line and relevant mechanisms. Toxicol. Appl. Pharmacol..

[103] Grabacka M., Plonka P.M., Urbanska K., Reiss K. (2006). Peroxisome proliferator-activated receptor alpha activation decreases metastatic potential of melanoma cells in vitro via down-regulation of Akt. Clin. Cancer Res..

[104] Chang N.-W., Wu C.-T., Chen D.-R., Yeh C.-Y., Lin C. (2013). High levels of arachidonic acid and peroxisome proliferator-activated receptor-alpha in breast cancer tissues are associated with promoting cancer cell proliferation. J. Nutr. Biochem..

[105] Cui J., Meng Q., Zhang X., Cui Q., Zhou W., Li S. (2015). Design and Synthesis of New α-Naphthoflavones as Cytochrome P450 (CYP) 1B1 Inhibitors To Overcome Docetaxel-Resistance Associated with CYP1B1 Overexpression. J. Med. Chem..

[106] Hwang Y.P., Won S.S., Jin S.W., Lee G.H., Pham T.H., Choi J.H., Kang K.W., Jeong H.G. (2019). WY-14643 Regulates CYP1B1 Expression through Peroxisome Proliferator-Activated Receptor α-Mediated Signaling in Human Breast Cancer Cells. Int. J. Mol. Sci..

[107] Castelli V., Catanesi M., Alfonsetti M., Laezza C., Lombardi F., Cinque B., Cifone M.G., Ippoliti R., Benedetti E., Cimini A. (2021). PPARα-Selective Antagonist GW6471 Inhibits Cell Growth in Breast Cancer Stem Cells Inducing Energy Imbalance and Metabolic Stress. Biomedicines.

[108] Pighetti G.M., Novosad W., Nicholson C., Hitt D.C., Hansens C., Hollingsworth A.B., Lerner M.L., Brackett D., Lightfoot S.A., Gimble J.M. (2001). Therapeutic treatment of DMBA-induced mammary tumors with PPAR ligands. Anticancer Res..

[109] Chandran K., Goswami S., Sharma-Walia N. (2016). Implications of a peroxisome proliferator-activated receptor alpha (PPARα) ligand clofibrate in breast cancer. Oncotarget.

[110] Yin X., Teng X., Ma T., Yang T., Zhang J., Huo M., Liu W., Yang Y., Yuan B., Yu H. (2022). RUNX2 recruits the NuRD(MTA1)/CRL4B complex to promote breast cancer progression and bone metastasis. Cell Death Differ..

[111] Shi Y., Hon M., Evans R.M. (2002). The peroxisome proliferator-activated receptor delta, an integrator of transcriptional repression and nuclear receptor signaling. Proc. Natl. Acad. Sci. USA.

[112] Schmidt A., Endo N., Rutledge S.J., Vogel R., Shinar D., Rodan G.A. (1992). Identification of a new member of the steroid hormone receptor superfamily that is activated by a peroxisome proliferator and fatty acids. Mol. Endocrinol..

[113] Amri E.Z., Bonino F., Ailhaud G., Abumrad N.A., Grimaldi P.A. (1995). Cloning of a protein that mediates transcriptional effects of fatty acids in preadipocytes. Homology to peroxisome proliferator-activated receptors. J. Biol. Chem..

[114] Evans R.M., Barish G.D., Wang Y.-X. (2004). PPARs and the complex journey to obesity. Nat. Med..

[115] Braissant O., Foufelle F., Scotto C., Dauça M., Wahli W. (1996). Differential expression of peroxisome proliferator-activated receptors (PPARs): Tissue distribution of PPAR-alpha, -beta, and -gamma in the adult rat. Endocrinology.

[116] Wang Y.-X., Lee C.-H., Tiep S., Yu R.T., Ham J., Kang H., Evans R.M. (2003). Peroxisome-proliferator-activated receptor delta activates fat metabolism to prevent obesity. Cell.

[117] Genini D., Catapano C.V. (2007). Block of nuclear receptor ubiquitination. A mechanism of ligand-dependent control of peroxisome proliferator-activated receptor delta activity. J. Biol. Chem..

[118] Wadosky K.M., Willis M.S. (2012). The story so far: Post-translational regulation of peroxisome proliferator-activated receptors by ubiquitination and SUMOylation. Am. J. Physiol. Heart Circ. Physiol..

[119] Rieck M., Wedeken L., Müller-Brüsselbach S., Meissner W., Müller R. (2007). Expression level and agonist-binding affect the turnover, ubiquitination and complex formation of peroxisome proliferator activated receptor beta. FEBS J..

[120] Sprecher D.L., Massien C., Pearce G., Billin A.N., Perlstein I., Willson T.M., Hassall D.G., Ancellin N., Patterson S.D., Lobe D.C. (2007). Triglyceride:high-density lipoprotein cholesterol effects in healthy subjects administered a peroxisome proliferator activated receptor delta agonist. Arterioscler. Thromb. Vasc. Biol..

[121] Tanaka T., Yamamoto J., Iwasaki S., Asaba H., Hamura H., Ikeda Y., Watanabe M., Magoori K., Ioka R.X., Tachibana K. (2003). Activation of peroxisome proliferator-activated receptor delta induces fatty acid beta-oxidation in skeletal muscle and attenuates metabolic syndrome. Proc. Natl. Acad. Sci. USA.

[122] de la Monte S.M., Wands J.R. (2008). Alzheimer’s disease is type 3 diabetes-evidence reviewed. J. Diabetes Sci. Technol..

[123] de la Monte S.M., Tong M., Lester-Coll N., Plater M.J., Wands J.R. (2006). Therapeutic rescue of neurodegeneration in experimental type 3 diabetes: Relevance to Alzheimer’s disease. J. Alzheimers Dis..

[124] Gross B., Hennuyer N., Bouchaert E., Rommens C., Grillot D., Mezdour H., Staels B. (2011). Generation and characterization of a humanized PPARδ mouse model. Br. J. Pharmacol..

[125] Liu S., Hatano B., Zhao M., Yen C.-C., Kang K., Reilly S.M., Gangl M.R., Gorgun C., Balschi J.A., Ntambi J.M. (2011). Role of peroxisome proliferator-activated receptor {delta}/{beta} in hepatic metabolic regulation. J. Biol. Chem..

[126] Shan W., Nicol C.J., Ito S., Bility M.T., Kennett M.J., Ward J.M., Gonzalez F.J., Peters J.M. (2008). Peroxisome proliferator-activated receptor-beta/delta protects against chemically induced liver toxicity in mice. Hepatology.

[127] Peters J.M., Gonzalez F.J. (2009). Sorting out the functional role(s) of peroxisome proliferator-activated receptor-beta/delta (PPARbeta/delta) in cell proliferation and cancer. Biochim. Biophys. Acta.

[128] Peters J.M., Hollingshead H.E., Gonzalez F.J. (2008). Role of peroxisome-proliferator-activated receptor beta/delta (PPARbeta/delta) in gastrointestinal tract function and disease. Clin. Sci..

[129] Tan N.S., Michalik L., Noy N., Yasmin R., Pacot C., Heim M., Flühmann B., Desvergne B., Wahli W. (2001). Critical roles of PPAR beta/delta in keratinocyte response to inflammation. Genes Dev..

[130] Schmuth M., Haqq C.M., Cairns W.J., Holder J.C., Dorsam S., Chang S., Lau P., Fowler A.J., Chuang G., Moser A.H. (2004). Peroxisome proliferator-activated receptor (PPAR)-beta/delta stimulates differentiation and lipid accumulation in keratinocytes. J. Investig. Dermatol..

[131] Kim D.J., Bility M.T., Billin A.N., Willson T.M., Gonzalez F.J., Peters J.M. (2006). PPARbeta/delta selectively induces differentiation and inhibits cell proliferation. Cell Death Differ..

[132] Romanowska M., Reilly L., Palmer C.N.A., Gustafsson M.C.U., Foerster J. (2010). Activation of PPARbeta/delta causes a psoriasis-like skin disease in vivo. PLoS ONE.

[133] Montagner A., Delgado M.B., Tallichet-Blanc C., Chan J.S.K., Sng M.K., Mottaz H., Degueurce G., Lippi Y., Moret C., Baruchet M. (2014). Src is activated by the nuclear receptor peroxisome proliferator-activated receptor β/δ in ultraviolet radiation-induced skin cancer. EMBO Mol. Med..

[134] Chong H.C., Tan M.J., Philippe V., Tan S.H., Tan C.K., Ku C.W., Goh Y.Y., Wahli W., Michalik L., Tan N.S. (2009). Regulation of epithelial-mesenchymal IL-1 signaling by PPARbeta/delta is essential for skin homeostasis and wound healing. J. Cell Biol..

[135] Yang L., Olsson B., Pfeifer D., Jönsson J.-I., Zhou Z.-G., Jiang X., Fredriksson B.-A., Zhang H., Sun X.-F. (2010). Knockdown of peroxisome proliferator-activated receptor-beta induces less differentiation and enhances cell-fibronectin adhesion of colon cancer cells. Oncogene.

[136] Marin H.E., Peraza M.A., Billin A.N., Willson T.M., Ward J.M., Kennett M.J., Gonzalez F.J., Peters J.M. (2006). Ligand activation of peroxisome proliferator-activated receptor beta inhibits colon carcinogenesis. Cancer Res..

[137] Santos G.C., Zielenska M., Prasad M., Squire J.A. (2007). Chromosome 6p amplification and cancer progression. J. Clin. Pathol..

[138] Uhlen M., Oksvold P., Fagerberg L., Lundberg E., Jonasson K., Forsberg M., Zwahlen M., Kampf C., Wester K., Hober S. (2010). Towards a knowledge-based Human Protein Atlas. Nat. Biotechnol..

[139] Berglund L., Björling E., Oksvold P., Fagerberg L., Asplund A., Szigyarto C.A.-K., Persson A., Ottosson J., Wernérus H., Nilsson P. (2008). A genecentric Human Protein Atlas for expression profiles based on antibodies. Mol. Cell. Proteomics.

[140] Uhlén M., Björling E., Agaton C., Szigyarto C.A.-K., Amini B., Andersen E., Andersson A.-C., Angelidou P., Asplund A., Asplund C. (2005). A human protein atlas for normal and cancer tissues based on antibody proteomics. Mol. Cell. Proteomics.

[141] Foreman J.E., Sharma A.K., Amin S., Gonzalez F.J., Peters J.M. (2010). Ligand activation of peroxisome proliferator-activated receptor-beta/delta (PPARbeta/delta) inhibits cell growth in a mouse mammary gland cancer cell line. Cancer Lett..

[142] Kittler R., Zhou J., Hua S., Ma L., Liu Y., Pendleton E., Cheng C., Gerstein M., White K.P. (2013). A comprehensive nuclear receptor network for breast cancer cells. Cell Rep..

[143] Stephen R.L., Gustafsson M.C.U., Jarvis M., Tatoud R., Marshall B.R., Knight D., Ehrenborg E., Harris A.L., Wolf C.R., Palmer C.N.A. (2004). Activation of peroxisome proliferator-activated receptor delta stimulates the proliferation of human breast and prostate cancer cell lines. Cancer Res..

[144] Girroir E.E., Hollingshead H.E., Billin A.N., Willson T.M., Robertson G.P., Sharma A.K., Amin S., Gonzalez F.J., Peters J.M. (2008). Peroxisome proliferator-activated receptor-beta/delta (PPARbeta/delta) ligands inhibit growth of UACC903 and MCF7 human cancer cell lines. Toxicology.

[145] Yao P.-L., Morales J.L., Zhu B., Kang B.-H., Gonzalez F.J., Peters J.M. (2014). Activation of peroxisome proliferator-activated receptor-β/δ (PPAR-β/δ) inhibits human breast cancer cell line tumorigenicity. Mol. Cancer Ther..

[146] Ghosh M., Ai Y., Narko K., Wang Z., Peters J.M., Hla T. (2009). PPARdelta is pro-tumorigenic in a mouse model of COX-2-induced mammary cancer. Prostaglandins Other Lipid Mediat..

[147] Yin Y., Russell R.G., Dettin L.E., Bai R., Wei Z.-L., Kozikowski A.P., Kopelovich L., Glazer R.I. (2005). Peroxisome proliferator-activated receptor delta and gamma agonists differentially alter tumor differentiation and progression during mammary carcinogenesis. Cancer Res..

[148] Pearce L.R., Komander D., Alessi D.R. (2010). The nuts and bolts of AGC protein kinases. Nat. Rev. Mol. Cell Biol..

[149] Pollock C.B., Yin Y., Yuan H., Zeng X., King S., Li X., Kopelovich L., Albanese C., Glazer R.I. (2011). PPARδ activation acts cooperatively with 3-phosphoinositide-dependent protein kinase-1 to enhance mammary tumorigenesis. PLoS ONE.

[150] Yuan H., Lu J., Xiao J., Upadhyay G., Umans R., Kallakury B., Yin Y., Fant M.E., Kopelovich L., Glazer R.I. (2013). PPARδ induces estrogen receptor-positive mammary neoplasia through an inflammatory and metabolic phenotype linked to mTOR activation. Cancer Res..

[151] Shearer B.G., Hoekstra W.J. (2003). Recent advances in peroxisome proliferator-activated receptor science. Curr. Med. Chem..

[152] Schug T.T., Berry D.C., Toshkov I.A., Cheng L., Nikitin A.Y., Noy N. (2008). Overcoming retinoic acid-resistance of mammary carcinomas by diverting retinoic acid from PPARbeta/delta to RAR. Proc. Natl. Acad. Sci. USA.

[153] Kannan-Thulasiraman P., Seachrist D.D., Mahabeleshwar G.H., Jain M.K., Noy N. (2010). Fatty acid-binding protein 5 and PPARbeta/delta are critical mediators of epidermal growth factor receptor-induced carcinoma cell growth. J. Biol. Chem..

[154] Borland M.G., Khozoie C., Albrecht P.P., Zhu B., Lee C., Lahoti T.S., Gonzalez F.J., Peters J.M. (2011). Stable over-expression of PPARβ/δ and PPARγ to examine receptor signaling in human HaCaT keratinocytes. Cell Signal..

[155] Borland M.G., Foreman J.E., Girroir E.E., Zolfaghari R., Sharma A.K., Amin S., Gonzalez F.J., Ross A.C., Peters J.M. (2008). Ligand activation of peroxisome proliferator-activated receptor-beta/delta inhibits cell proliferation in human HaCaT keratinocytes. Mol. Pharmacol..

[156] Wang X., Wang G., Shi Y., Sun L., Gorczynski R., Li Y.-J., Xu Z., Spaner D.E. (2016). PPAR-delta promotes survival of breast cancer cells in harsh metabolic conditions. Oncogenesis.

[157] Burdick A.D., Bility M.T., Girroir E.E., Billin A.N., Willson T.M., Gonzalez F.J., Peters J.M. (2007). Ligand activation of peroxisome proliferator-activated receptor-beta/delta(PPARbeta/delta) inhibits cell growth of human N/TERT-1 keratinocytes. Cell Signal..

[158] Adhikary T., Brandt D.T., Kaddatz K., Stockert J., Naruhn S., Meissner W., Finkernagel F., Obert J., Lieber S., Scharfe M. (2013). Inverse PPARβ/δ agonists suppress oncogenic signaling to the ANGPTL4 gene and inhibit cancer cell invasion. Oncogene.

[159] Gimble J.M., Pighetti G.M., Lerner M.R., Wu X., Lightfoot S.A., Brackett D.J., Darcy K., Hollingsworth A.B. (1998). Expression of peroxisome proliferator activated receptor mRNA in normal and tumorigenic rodent mammary glands. Biochem. Biophys. Res. Commun..

[160] Fajas L., Auboeuf D., Raspé E., Schoonjans K., Lefebvre A.M., Saladin R., Najib J., Laville M., Fruchart J.C., Deeb S. (1997). The organization, promoter analysis, and expression of the human PPARgamma gene. J. Biol. Chem..

[161] Fang S., Livergood M.C., Nakagawa P., Wu J., Sigmund C.D. (2021). Role of the Peroxisome Proliferator Activated Receptors in Hypertension. Circ. Res..

[162] Francque S., Szabo G., Abdelmalek M.F., Byrne C.D., Cusi K., Dufour J.-F., Roden M., Sacks F., Tacke F. (2021). Nonalcoholic steatohepatitis: The role of peroxisome proliferator-activated receptors. Nat. Rev. Gastroenterol. Hepatol..

[163] Azhar S. (2010). Peroxisome proliferator-activated receptors, metabolic syndrome and cardiovascular disease. Future Cardiol..

[164] Takeyama K., Kodera Y., Suzawa M., Kato S. (2000). [Peroxisome proliferator-activated receptor(PPAR)--structure, function, tissue distribution, gene expression]. Nihon Rinsho..

[165] Janani C., Ranjitha Kumari B.D. (2015). PPAR gamma gene--a review. Diabetes Metab. Syndr..

[166] Hamblin M., Chang L., Fan Y., Zhang J., Chen Y.E. (2009). PPARs and the cardiovascular system. Antioxid. Redox Signal..

[167] Li Y., Qi Y., Huang T.H.W., Yamahara J., Roufogalis B.D. (2008). Pomegranate flower: A unique traditional antidiabetic medicine with dual PPAR-alpha/-gamma activator properties. Diabetes. Obes. Metab..

[168] Blaschke F., Caglayan E., Hsueh W.A. (2006). Peroxisome proliferator-activated receptor gamma agonists: Their role as vasoprotective agents in diabetes. Endocrinol. Metab. Clin. N. Am..

[169] Rizos C.V., Kei A., Elisaf M.S. (2016). The current role of thiazolidinediones in diabetes management. Arch. Toxicol..

[170] Kahn C.R., Chen L., Cohen S.E. (2000). Unraveling the mechanism of action of thiazolidinediones. J. Clin. Investig..

[171] Hauner H. (2002). The mode of action of thiazolidinediones. Diabetes. Metab. Res. Rev..

[172] Farshbaf M.J., Ghaedi K., Shirani M., Nasr-Esfahani M.H. (2014). Peroxisome proliferator activated receptor gamma (PPARγ) as a therapeutic target for improvement of cognitive performance in Fragile-X. Med. Hypotheses.

[173] Yang Y., Zhao L.-H., Huang B., Wang R.-Y., Yuan S.-X., Tao Q.-F., Xu Y., Sun H.-Y., Lin C., Zhou W.-P. (2015). Pioglitazone, a PPARγ agonist, inhibits growth and invasion of human hepatocellular carcinoma via blockade of the rage signaling. Mol. Carcinog..

[174] Wang Y., Tan H., Xu D., Ma A., Zhang L., Sun J., Yang Z., Liu Y., Shi G. (2014). The combinatory effects of PPAR-γ agonist and survivin inhibition on the cancer stem-like phenotype and cell proliferation in bladder cancer cells. Int. J. Mol. Med..

[175] Velmurugan B.K., Yang H.-H., Sung P.-J., Weng C.-F. (2017). Excavatolide B inhibits nonsmall cell lung cancer proliferation by altering peroxisome proliferator activated receptor gamma expression and PTEN/AKT/NF-Kβ expression. Environ. Toxicol..

[176] Srivastava N., Kollipara R.K., Singh D.K., Sudderth J., Hu Z., Nguyen H., Wang S., Humphries C.G., Carstens R., Huffman K.E. (2014). Inhibition of cancer cell proliferation by PPARγ is mediated by a metabolic switch that increases reactive oxygen species levels. Cell Metab..

[177] Pestereva E., Kanakasabai S., Bright J.J. (2012). PPARγ agonists regulate the expression of stemness and differentiation genes in brain tumour stem cells. Br. J. Cancer.

[178] Trindade-da-Silva C.A., Reis C.F., Vecchi L., Napimoga M.H., Sperandio M., Matias Colombo B.F., Alves P.T., Ward L.S., Ueira-Vieira C., Goulart L.R. (2016). 15-Deoxy-Δ(12,14)-prostaglandin J2 Induces Apoptosis and Upregulates SOCS3 in Human Thyroid Cancer Cells. PPAR Res..

[179] Wu K., Yang Y., Liu D., Qi Y., Zhang C., Zhao J., Zhao S. (2016). Activation of PPARγ suppresses proliferation and induces apoptosis of esophageal cancer cells by inhibiting TLR4-dependent MAPK pathway. Oncotarget.

[180] Zurlo D., Ziccardi P., Votino C., Colangelo T., Cerchia C., Dal Piaz F., Dallavalle S., Moricca S., Novellino E., Lavecchia A. (2016). The antiproliferative and proapoptotic effects of cladosporols A and B are related to their different binding mode as PPARγ ligands. Biochem. Pharmacol..

[181] Weidner C., Rousseau M., Micikas R.J., Fischer C., Plauth A., Wowro S.J., Siems K., Hetterling G., Kliem M., Schroeder F.C. (2016). Amorfrutin C Induces Apoptosis and Inhibits Proliferation in Colon Cancer Cells through Targeting Mitochondria. J. Nat. Prod..

[182] Kohno H., Yasui Y., Suzuki R., Hosokawa M., Miyashita K., Tanaka T. (2004). Dietary seed oil rich in conjugated linolenic acid from bitter melon inhibits azoxymethane-induced rat colon carcinogenesis through elevation of colonic PPARgamma expression and alteration of lipid composition. Int. J. Cancer.

[183] Yasui Y., Hosokawa M., Sahara T., Suzuki R., Ohgiya S., Kohno H., Tanaka T., Miyashita K. (2005). Bitter gourd seed fatty acid rich in 9c,11t,13t-conjugated linolenic acid induces apoptosis and up-regulates the GADD45, p53 and PPARgamma in human colon cancer Caco-2 cells. Prostaglandins. Leukot. Essent. Fatty Acids.

[184] Mueller E., Sarraf P., Tontonoz P., Evans R.M., Martin K.J., Zhang M., Fletcher C., Singer S., Spiegelman B.M. (1998). Terminal differentiation of human breast cancer through PPAR gamma. Mol. Cell.

[185] Moon H.-S., Guo D.-D., Lee H.-G., Choi Y.-J., Kang J.-S., Jo K., Eom J.-M., Yun C.-H., Cho C.-S. (2010). Alpha-eleostearic acid suppresses proliferation of MCF-7 breast cancer cells via activation of PPARgamma and inhibition of ERK 1/2. Cancer Sci..

[186] Bonofiglio D., Gabriele S., Aquila S., Catalano S., Gentile M., Middea E., Giordano F., Andò S. (2005). Estrogen receptor alpha binds to peroxisome proliferator-activated receptor response element and negatively interferes with peroxisome proliferator-activated receptor gamma signaling in breast cancer cells. Clin. Cancer Res..

[187] Bonofiglio D., Aquila S., Catalano S., Gabriele S., Belmonte M., Middea E., Qi H., Morelli C., Gentile M., Maggiolini M. (2006). Peroxisome proliferator-activated receptor-gamma activates p53 gene promoter binding to the nuclear factor-kappaB sequence in human MCF7 breast cancer cells. Mol. Endocrinol..

[188] Bonofiglio D., Gabriele S., Aquila S., Qi H., Belmonte M., Catalano S., Andò S. (2009). Peroxisome proliferator-activated receptor gamma activates fas ligand gene promoter inducing apoptosis in human breast cancer cells. Breast Cancer Res. Treat..

[189] Chinnaiyan A.M., Dixit V.M. (1997). Portrait of an executioner: The molecular mechanism of FAS/APO-1-induced apoptosis. Semin. Immunol..

[190] Debatin K.-M. (2004). Apoptosis pathways in cancer and cancer therapy. Cancer Immunol. Immunother..

[191] Pinkoski M.J., Green D.R. (1999). Fas ligand, death gene. Cell Death Differ..

[192] Catalano S., Mauro L., Bonofiglio D., Pellegrino M., Qi H., Rizza P., Vizza D., Bossi G., Andò S. (2011). In vivo and in vitro evidence that PPARγ ligands are antagonists of leptin signaling in breast cancer. Am. J. Pathol..

[193] Burstein H.J., Demetri G.D., Mueller E., Sarraf P., Spiegelman B.M., Winer E.P. (2003). Use of the peroxisome proliferator-activated receptor (PPAR) gamma ligand troglitazone as treatment for refractory breast cancer: A phase II study. Breast Cancer Res. Treat..

[194] Gale E.A. (2001). Lessons from the glitazones: A story of drug development. Lancet.

[195] Krentz A.J., Bailey C.J. (2005). Oral antidiabetic agents: Current role in type 2 diabetes mellitus. Drugs.

[196] Garcia-Vallvé S., Guasch L., Tomas-Hernández S., del Bas J.M., Ollendorff V., Arola L., Pujadas G., Mulero M. (2015). Peroxisome Proliferator-Activated Receptor γ (PPARγ) and Ligand Choreography: Newcomers Take the Stage. J. Med. Chem..

[197] Yee L.D., Williams N., Wen P., Young D.C., Lester J., Johnson M.V., Farrar W.B., Walker M.J., Povoski S.P., Suster S. (2007). Pilot study of rosiglitazone therapy in women with breast cancer: Effects of short-term therapy on tumor tissue and serum markers. Clin Cancer Res..

[198] Elstner E., Müller C., Koshizuka K., Williamson E.A., Park D., Asou H., Shintaku P., Said J.W., Heber D., Koeffler H.P. (1998). Ligands for peroxisome proliferator-activated receptorgamma and retinoic acid receptor inhibit growth and induce apoptosis of human breast cancer cells in vitro and in BNX mice. Proc. Natl. Acad. Sci. USA.

[199] Mustafa A., Kruger W.D. (2008). Suppression of tumor formation by a cyclooxygenase-2 inhibitor and a peroxisome proliferator-activated receptor gamma agonist in an in vivo mouse model of spontaneous breast cancer. Clin. Cancer Res..

[200] Bonofiglio D., Cione E., Qi H., Pingitore A., Perri M., Catalano S., Vizza D., Panno M.L., Genchi G., Fuqua S.A.W. (2009). Combined low doses of PPARgamma and RXR ligands trigger an intrinsic apoptotic pathway in human breast cancer cells. Am. J. Pathol..

[201] Yamazaki K., Shimizu M., Okuno M., Matsushima-Nishiwaki R., Kanemura N., Araki H., Tsurumi H., Kojima S., Weinstein I.B., Moriwaki H. (2007). Synergistic effects of RXR alpha and PPAR gamma ligands to inhibit growth in human colon cancer cells--phosphorylated RXR alpha is a critical target for colon cancer management. Gut.

[202] Jiang Y., Huang Y., Cheng C., Lu W., Zhang Y., Liu X., Zou L., Ben Q., Shen A. (2011). Combination of thiazolidinedione and hydralazine suppresses proliferation and induces apoptosis by PPARγ up-expression in MDA-MB-231 cells. Exp. Mol. Pathol..

[203] Girnun G.D., Naseri E., Vafai S.B., Qu L., Szwaya J.D., Bronson R., Alberta J.A., Spiegelman B.M. (2007). Synergy between PPARgamma ligands and platinum-based drugs in cancer. Cancer Cell.

[204] Bräutigam K., Biernath-Wüpping J., Bauerschlag D.O., von Kaisenberg C.S., Jonat W., Maass N., Arnold N., Meinhold-Heerlein I. (2011). Combined treatment with TRAIL and PPARγ ligands overcomes chemoresistance of ovarian cancer cell lines. J. Cancer Res. Clin. Oncol..

[205] Yokoyama Y., Xin B., Shigeto T., Mizunuma H. (2011). Combination of ciglitazone, a peroxisome proliferator-activated receptor gamma ligand, and cisplatin enhances the inhibition of growth of human ovarian cancers. J. Cancer Res. Clin. Oncol..

[206] Cesario R.M., Stone J., Yen W.-C., Bissonnette R.P., Lamph W.W. (2006). Differentiation and growth inhibition mediated via the RXR:PPARgamma heterodimer in colon cancer. Cancer Lett..

[207] Desreumaux P., Dubuquoy L., Nutten S., Peuchmaur M., Englaro W., Schoonjans K., Derijard B., Desvergne B., Wahli W., Chambon P. (2001). Attenuation of colon inflammation through activators of the retinoid X receptor (RXR)/peroxisome proliferator-activated receptor gamma (PPARgamma) heterodimer. A basis for new therapeutic strategies. J. Exp. Med..

[208] Fu H., Zhang J., Pan J., Zhang Q., Lu Y., Wen W., Lubet R.A., Szabo E., Chen R., Wang Y. (2011). Chemoprevention of lung carcinogenesis by the combination of aerosolized budesonide and oral pioglitazone in A/J mice. Mol. Carcinog..

[209] Reddy R.C., Srirangam A., Reddy K., Chen J., Gangireddy S., Kalemkerian G.P., Standiford T.J., Keshamouni V.G. (2008). Chemotherapeutic drugs induce PPAR-gamma expression and show sequence-specific synergy with PPAR-gamma ligands in inhibition of non-small cell lung cancer. Neoplasia.

[210] Aronica S.M., Kraus W.L., Katzenellenbogen B.S. (1994). Estrogen action via the cAMP signaling pathway: Stimulation of adenylate cyclase and cAMP-regulated gene transcription. Proc. Natl. Acad. Sci. USA.

[211] Migliaccio A., Di Domenico M., Castoria G., de Falco A., Bontempo P., Nola E., Auricchio F. (1996). Tyrosine kinase/p21ras/MAP-kinase pathway activation by estradiol-receptor complex in MCF-7 cells. EMBO J..

[212] Keller H., Givel F., Perroud M., Wahli W. (1995). Signaling cross-talk between peroxisome proliferator-activated receptor/retinoid X receptor and estrogen receptor through estrogen response elements. Mol. Endocrinol..

[213] Cui Y., Miyoshi K., Claudio E., Siebenlist U.K., Gonzalez F.J., Flaws J., Wagner K.-U., Hennighausen L. (2002). Loss of the peroxisome proliferation-activated receptor gamma (PPARgamma ) does not affect mammary development and propensity for tumor formation but leads to reduced fertility. J. Biol. Chem..

[214] Yin Y., Yuan H., Zeng X., Kopelovich L., Glazer R.I. (2009). Inhibition of peroxisome proliferator-activated receptor gamma increases estrogen receptor-dependent tumor specification. Cancer Res..

[215] Saez E., Rosenfeld J., Livolsi A., Olson P., Lombardo E., Nelson M., Banayo E., Cardiff R.D., Izpisua-Belmonte J.C., Evans R.M. (2004). PPAR gamma signaling exacerbates mammary gland tumor development. Genes Dev..

[216] Tian L., Zhou J., Casimiro M.C., Liang B., Ojeifo J.O., Wang M., Hyslop T., Wang C., Pestell R.G. (2009). Activating peroxisome proliferator-activated receptor gamma mutant promotes tumor growth in vivo by enhancing angiogenesis. Cancer Res..

[217] Xu Y.-Y., Liu H., Su L., Xu N., Xu D.-H., Liu H.-Y., Spaner D., Bed-David Y., Li Y.-J. (2019). PPARγ inhibits breast cancer progression by upregulating PTPRF expression. Eur. Rev. Med. Pharmacol. Sci..

[218] Kotta-Loizou I., Giaginis C., Theocharis S. (2012). The role of peroxisome proliferator-activated receptor-γ in breast cancer. Anticancer. Agents Med. Chem..

[219] Yang P.-B., Hou P.-P., Liu F.-Y., Hong W.-B., Chen H.-Z., Sun X.-Y., Li P., Zhang Y., Ju C.-Y., Luo L.-J. (2020). Blocking PPARγ interaction facilitates Nur77 interdiction of fatty acid uptake and suppresses breast cancer progression. Proc. Natl. Acad. Sci. USA.

[220] Papadaki I., Mylona E., Giannopoulou I., Markaki S., Keramopoulos A., Nakopoulou L. (2005). PPARgamma expression in breast cancer: Clinical value and correlation with ERbeta. Histopathology.

[221] Kulkarni S., Patil D.B., Diaz L.K., Wiley E.L., Morrow M., Khan S.A. (2008). COX-2 and PPARgamma expression are potential markers of recurrence risk in mammary duct carcinoma in-situ. BMC Cancer.

[222] Liedtke C., Mazouni C., Hess K.R., André F., Tordai A., Mejia J.A., Symmans W.F., Gonzalez-Angulo A.M., Hennessy B., Green M. (2008). Response to neoadjuvant therapy and long-term survival in patients with triple-negative breast cancer. J. Clin. Oncol..

[223] Li T., Zhang Q., Zhang J., Yang G., Shao Z., Luo J., Fan M., Ni C., Wu Z., Hu X. (2014). Fenofibrate induces apoptosis of triple-negative breast cancer cells via activation of NF-κB pathway. BMC Cancer..

[224] Kwong S.C., Jamil A.H.A., Rhodes A., Taib N.A., Chung I. (2019). Metabolic role of fatty acid binding protein 7 in mediating triple-negative breast cancer cell death via PPAR-α signaling. J. Lipid Res..

[225] Apaya M.K., Hsiao P.W., Yang Y.C., Shyur L.F. (2020). Deregulating the CYP2C19/Epoxy-Eicosatrienoic Acid-Associated FABP4/FABP5 Signaling Network as a Therapeutic Approach for Metastatic Triple-Negative Breast Cancer. Cancers.

[226] Volkers N. (2000). Diabetes and cancer: Scientists search for a possible link. J. Natl. Cancer Inst..

[227] Giovannucci E., Harlan D.M., Archer M.C., Bergenstal R.M., Gapstur S.M., Habel L.A., Pollak M., Regensteiner J.G., Yee D. (2010). Diabetes and cancer: A consensus report. Diabetes Care.

[228] Rossouw J.E., Anderson G.L., Prentice R.L., LaCroix A.Z., Kooperberg C., Stefanick M.L., Jackson R.D., Beresford S.A.A., Howard B.V., Johnson K.C. (2002). Risks and benefits of estrogen plus progestin in healthy postmenopausal women: Principal results From the Women’s Health Initiative randomized controlled trial. JAMA.

[229] Youssef J., Badr M. (2011). Peroxisome proliferator-activated receptors and cancer: Challenges and opportunities. Br. J. Pharmacol..

